# Towards Dual-Tracer SPECT for Prostate Cancer Imaging Using [^99m^Tc]Tc-PSMA-I&S and [^111^In]In-RM2

**DOI:** 10.3390/ph18071002

**Published:** 2025-07-03

**Authors:** Carolina Giammei, Theresa Balber, Veronika Felber, Thomas Dillinger, Jens Cardinale, Marie R. Brandt, Anna Stingeder, Markus Mitterhauser, Gerda Egger, Thomas L. Mindt

**Affiliations:** 1 Ludwig Boltzmann Institute Applied Diagnostics, General Hospital of Vienna, c/o Sekretariat Nuklearmedizin, Währiger Gürtel 18-20, 1090 Vienna, Austria; carolina.giammei@lbiad.lbg.ac.at (C.G.); theresa.balber@meduniwien.ac.at (T.B.); vroni.felber@tum.de (V.F.); thomas.dillinger@lbiad.lbg.ac.at (T.D.); jens.cardinale@lbiad.lbg.ac.at (J.C.); marie.brandt@meduniwien.ac.at (M.R.B.); anna.stingeder@lbiad.lbg.ac.at (A.S.); markus.mitterhauser@meduniwien.ac.at (M.M.); gerda.egger@meduniwien.ac.at (G.E.); 2 Institute of Inorganic Chemistry, Faculty of Chemistry, University of Vienna, Währinger Straße 42, 1090 Vienna, Austria; 3 Division of Nuclear Medicine, Department of Biomedical Imaging and Image-Guided Therapy, Medical University of Vienna, Währinger Gürtel 18-20, 1090 Vienna, Austria; 4 Vienna Doctoral School in Chemistry, University of Vienna, Währinger Straße 42, 1090 Vienna, Austria; 5 Joint Applied Medicinal Radiochemistry Facility, University of Vienna and Medical University of Vienna, 1090 Vienna, Austria; 6 Department of Pathology, Medical University of Vienna, Währinger Gürtel 18-20, 1090 Vienna, Austria

**Keywords:** dual-tracer approach, indium-111, technetium-99m, SPECT, GRPR, PSMA, tumor heterogeneity, CHO-K1-GRPR, CHO-K1-PSMA

## Abstract

**Background/Objectives**: Radiolabeled biomolecules specifically targeting overexpressed structures on tumor cells hold great potential for prostate cancer (PCa) imaging and therapy. Due to heterogeneous target expression, single radiopharmaceuticals may not detect or treat all lesions, while simultaneously applying two or more radiotracers potentially improves staging, stratification, and therapy of cancer patients. This study explores a dual-tracer SPECT approach using [^111^In]In-RM2 (targeting the gastrin-releasing peptide receptor, GRPR) and [^99m^Tc]Tc-PSMA-I&S (targeting the prostate-specific membrane antigen, PSMA) as a proof of concept. To mimic heterogeneous tumor lesions in the same individual, we aimed to establish a dual xenograft mouse model for preclinical evaluation. **Methods**: CHO-K1 cells underwent lentiviral transduction for human GRPR or human PSMA overexpression. Six-to-eight-week-old female immunodeficient mice (NOD SCID) were subsequently inoculated with transduced CHO-K1 cells in both flanks, enabling a dual xenograft with similar target density and growth of both xenografts. Respective dual-isotope imaging and γ-counting protocols were established. Target expression was analyzed *ex vivo* by Western blotting. **Results**: *In vitro* studies showed similar target-specific binding and internalization of [^111^In]In-RM2 and [^99m^Tc]Tc-PSMA-I&S in transduced CHO-K1 cells compared to reference lines PC-3 and LNCaP. However, *in vivo* imaging showed negligible tumor uptake in xenografts of the transduced cell lines. *Ex vivo* analysis indicated a loss of the respective biomarkers in the xenografts. **Conclusions**: Although the technical feasibility of a dual-tracer SPECT imaging approach using ^111^In and ^99m^Tc has been demonstrated, the potential of [^99m^Tc]Tc-PSMA-I&S and [^111^In]In-RM2 in a dual-tracer cocktail to improve PCa diagnosis could not be verified. The animal model, and in particular the transduced cell lines developed exclusively for this project, proved to be unsuitable for this purpose. The *in*/*ex vivo* experiments indicated that results from an *in vitro* model may not necessarily be successfully transferred to an *in vivo* setting. To assess the potential of this dual-tracer concept to improve PCa diagnosis, optimized *in vivo* models are needed. Nevertheless, our strategies address key challenges in dual-tracer applications, aiming to optimize future SPECT imaging approaches.

## 1. Introduction

Prostate cancer (PCa) represents a relevant cause of morbidity and mortality affecting men worldwide. The great spatial and morphological heterogeneity, as well as the genetic diversity of PCa, outline the complexity of this disease in the staging and treatment of patients [1,2]. Molecular imaging of PCa with radiopharmaceuticals assesses the increased metabolic rate of cancer cells, the tumor-specific expression of receptors and membrane proteins, or osteoblastic signaling pathways adjacent to bone metastases [3]. The prostate-specific membrane antigen (PSMA), also known as glutamate carboxypeptidase II (GCP II), is a type II transmembrane glycoprotein encoded by the FOLH1 gene. Overexpression of PSMA on PCa cells is associated with higher prostate-specific antigen (PSA) levels, tumor progression (hormone-refractory and metastatic PCa), and low overall survival [4,5]. However, PSMA expression is upregulated in some but not in all PCa patients and thus demonstrates a remarkable inter- and intra-patient heterogeneity [6]. Despite the great success of radiolabeled PSMA ligands, especially in the detection of metastatic PCa, interpretation of PSMA-based images is associated with some pitfalls [7]. Tumors can grow in cell subpopulations, which can become tolerant to pharmaceutical treatments. The factors that lead to tumor heterogeneity during PCa progression are still not fully understood, and therefore, there is a need to develop more suitable imaging agents and/or techniques in order to better understand the molecular pathways associated with phenotype transformations of PCa [8]. Radiolabeled target-specific biomolecules hold a great potential in nuclear medicine for the imaging and therapy of PCa [9] and could also serve as valuable tools to investigate spatial and temporal tumor heterogeneity. Despite the outstanding performance of radiolabeled PSMA ligands [10,11], the detection and treatment of certain PCa lesions is not possible due to relatively low radioligand uptake. PSMA expression is absent or insufficient in about 10% of primary PCa, in neuroendocrine-differentiated PCa, and in lesions smaller than 5 mm [12]. Since the treatment and management of PCa patients depends on the outcome from staging and re-staging, radiopharmaceuticals able to target other biological processes of PCa are needed in order to improve the diagnostic accuracy [12].

Another relevant biomarker for PCa is the gastrin-releasing peptide receptor (GRPR), which has been reported to be expressed primarily in treatment-naïve PCa and during early stages of disease [13,14]. Since different biological processes regulate the expression of PSMA and GRPR, understanding the role of these two tumor biomarkers in patients with PCa could prove helpful for a better management of the disease.

In a direct comparison, [^68^Ga]Ga-PSMA-11 and [^68^Ga]Ga-RM2 displayed distinctly different biodistribution patterns and uptake in suspected lesions in a small cohort of patients with biochemically recurrent PCa [15]. This difference is supposed to originate from heterogeneity of the lesions, and some patients might benefit from imaging using both agents to adjust the therapy regimen accordingly (e.g., GRPR-targeted radioligand therapy (RLT) instead of PSMA-targeted RLT). The reported heterogeneous expression of these two biomarkers led to the development of bispecific heterodimers, targeting both GRPR and PSMA [12,16,17,18]. While these approaches have great potential to achieve increased tumor avidity and improved sensitivity (e.g., more lesions detectable), they do not allow for the separate identification, localization, and quantification of lesions with different expression patterns of the biomarkers. In contrast, dual-tracer SPECT could provide more valuable information to nuclear medicine physicians and realize a more personalized and effective treatment for the patient. This non-invasive method allows identification and localization of intrapatient and intratumoral heterogeneity and ideally enables quantification of lesions with different biomarker expression. Therefore, it is supposed to support physicians in disease assessment and adaptation of the treatment regimen for the patient accordingly.

**Figure 1:**
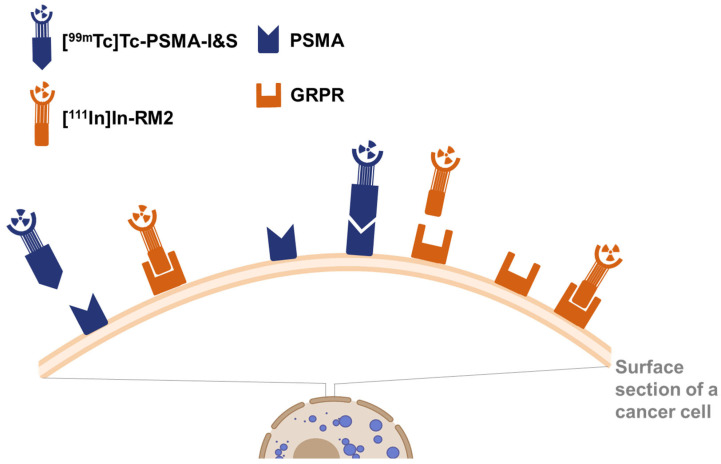
Graphical representation of the dual-tracer imaging concept, planned to be realized by simultaneous application of differently labeled radiotracers targeting PSMA or GRPR.

Radiotracers labeled with different isotopes can be co-injected and detected separately, provided that isotopes with distinctly different emission energies are selected (Figure 1). Dual-isotope (DI) SPECT imaging has been clinically used for myocardial perfusion imaging [19,20], for hyperparathyroidism imaging [21], for the detection of early disease spread in patients with prostate cancer [22], and for the determination of the ratio of serotonin and dopamine transporters in depression [23] among other applications. Preclinical research focused on dual-isotope SPECT approaches to identify hepatic lesions [24], to quantify the beta cell mass in rodents [25], and to directly compare the biodistribution of radioligands in the same animal [26]. The aim of this study was to provide a preclinical proof of principle of a DI SPECT approach using Technetium-99m (^99m^Tc) and Indium-111 (^111^In) for the separate detection of either PSMA- or GRPR-positive lesions.

## 2. Results

### 2.1. Syntheses and Radiolabeling

#### 2.1.1. RM2 Synthesis and Radiolabeling

The synthesis of RM2 was achieved by manual solid-phase peptide synthesis (SPPS), leading to the final peptide in its free chelator form in a chemical purity of >95% (SI, Figures S1–S3). RM2 was radiolabeled with ^111^In achieving a radiochemical purity of ≥95% as determined by analytical radio-RP-HPLC (Supporting Information, SI, Figure S15A). The identity of [^111^In]In-RM2 was confirmed by injection of the non-radioactive analogue ^nat^In-RM2 at the same HPLC solvent gradient as for [^111^In]In-RM2 (SI, Figure S15B). Synthesis of ^nat^In-RM2 is described in the SI (Section 3.1.2).

#### 2.1.2. PSMA-I&S Synthesis and Radiolabeling

PSMA-I&S was assembled by manual SPPS and fragment condensation, resulting in chemical purities > 99% (SI, Scheme S1, Figures S4–S14). Radiolabeling of PSMA-I&S was performed with [^99m^Tc]TcO_4_^-^ in a kit-based reaction procedure, and the final radioligand [^99m^Tc]Tc-PSMA-I&S was obtained in a radiochemical purity of ≥95% as determined by analytical radio-RP-HPLC (SI, Figure S16).

**Figure 2:**
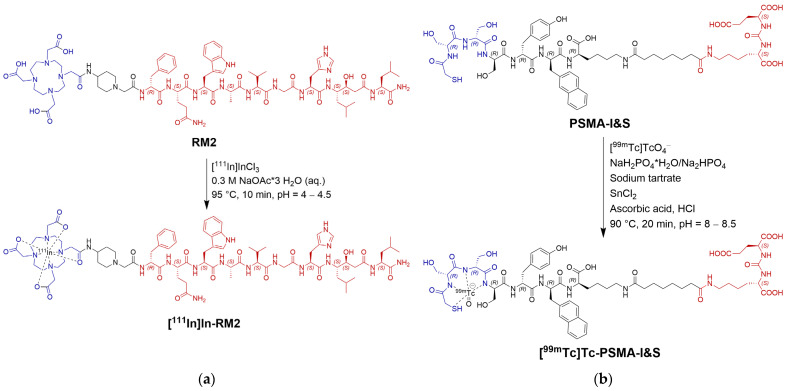
Structure of (**a**) RM2 and (**b**) PSMA-I&S with reaction schemes leading to the final radiolabeled peptides. Indicated in red are the binding motifs for GRPR or PSMA, respectively. Chelators for radiometal complexation are displayed in blue.

General procedures for ^111^In-labeling of RM2 as well as for ^99m^Tc-labeling of PSMA-I&S are displayed in Figure 2.

### 2.2. CHO-K1 Cell Transduction with Human GRPR or Human PSMA

**Figure 3:**
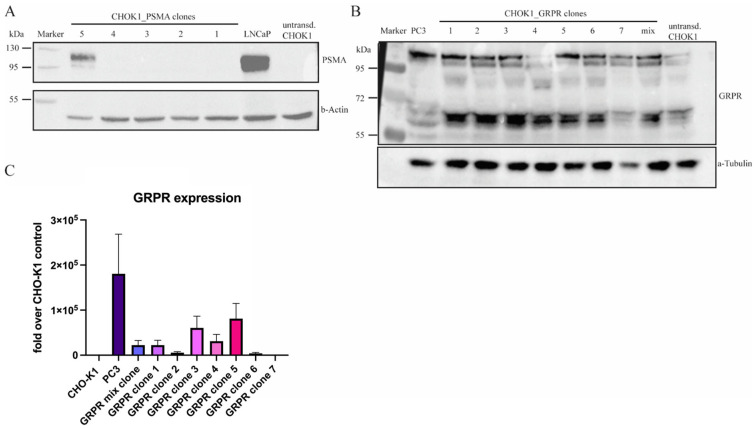
Confirmation of PSMA and GRPR expression in CHO-K1 cells. CHO-K1 cells underwent lentiviral transduction for PSMA or GRPR overexpression. Selected clones were picked for Western blot analysis to investigate (**A**) PSMA expression in CHO-K1 single clones. PSMA^+^ human LNCaP prostate cancer cells were used as positive control and β-Actin was used as loading control. (**B**) For GRPR, overexpression in selected CHO-K1-GRPR clones was shown by using an anti-GRPR antibody targeting an extracellular domain of GRPR (Alomone labs, ABR 002), GRPR^+^ human PC-3 prostate cancer cells were used as positive control, and α-Tubulin was used as loading control. Bands of various sizes represent the differently glycosylated forms of the GRPR protein. (**C**) In addition to Western blots, GRPR mRNA expression was measured by using RT-qPCR for human GRPR gene expression. Fold change was calculated over non-transduced CHO-K1 cells. PC-3 cells were used as a positive control and β-Actin was used as the reference gene.

Overexpression of both human GRPR and PSMA in single clones of transduced CHO-K1 cells was confirmed by Western blot analysis (Figure 3). Since non-transduced CHO-K1 cells expressed low levels of the homologous hamster GRPR receptor, we additionally confirmed the expression of human *GRPR* for CHO-K1-GRPR-transduced clones by RT-qPCR. Based on these results, CHO-K1-PSMA clone 5 and CHO-K1-GRPR clone 3 were chosen for further experiments.

### 2.3. In Vitro Evaluation

For in vitro evaluation of [^111^In]In-RM2 and [^99m^Tc]Tc-PSMA-I&S, CHO-K1-GRPR (clone 3) as well as CHO-K1-PSMA (clone 5) cells were used, which stably express the human GRPR or PSMA protein, respectively.

**Figure 4:**
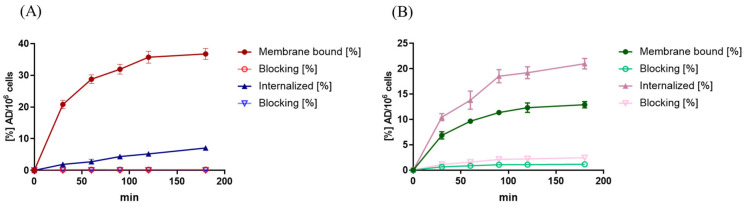
Membrane-bound and internalized fraction of (**A**) [^111^In]In-RM2 and (**B**) [^99m^Tc]Tc-PSMA-I&S using CHO-K1-GRPR and CHO-K1-PSMA cells, respectively. Non-specific binding was determined with 1000-fold excess of Bombesin (BBN) (1–14) for [^111^In]In-RM2 and 1000-fold excess of 2-phosphonomethyl pentanedioic acid (2-PMPA) for [^99m^Tc]Tc-PSMA-I&S (‘Blocking’). Data points show mean values ± SD (*n* = 3 in triplicate); data are normalized to 10^6^ cells per well.

Membrane-binding equilibrium was reached after approx. 2 h of incubation for both radiolabeled compounds, [^111^In]In-RM2 and [^99m^Tc]Tc-PSMA-I&S. At this time point, the values for internalized and membrane-bound fraction (=total uptake) accounted for 40.9% ± 2.4% and 31.5% ± 1.9% of the applied dose (AD) per 10^6^ cells for [^111^In]In-RM2 and [^99m^Tc]Tc-PSMA-I&S, respectively (not corrected for non-specific binding), while non-specific uptake (‘Blocking’ condition) was low (Figure 4A,B, Table 1).

**Figure 5:**
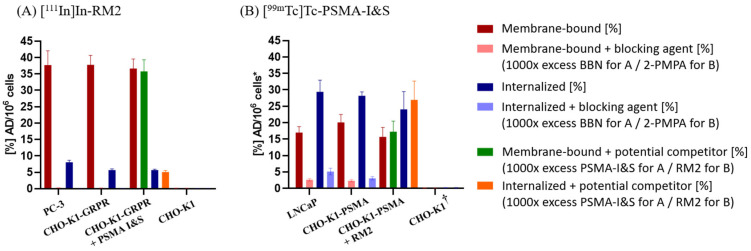
Single-time point (2 h) internalization assay with (**A**) [^111^In]In-RM2 using PC-3 (positive control), CHO-K1-GRPR and CHO-K1 cells (negative control); (**B**) [^99m^Tc]Tc-PSMA-I&S using LNCaP (positive control), CHO-K1-PSMA and CHO-K1 cells (negative control). Non-specific binding was determined with 1000-fold excess of BBN(1–14) or 2-PMPA for [^111^In]In-RM2 and [^99m^Tc]Tc-PSMA-I&S, respectively (some of these values are very close to zero and thus not visible in the graph but displayed in the SI, Figure S17). Cross-validation experiments with 1000-fold excess of PSMA-I&S or RM2 (potential competitors) are also displayed (green- and orange-colored bars). Data points show mean values ± SD (*n* = 3 and ^†^ *n* = 2 in triplicate). Data are normalized to 10^6^ cells per well; * LNCaP values are measured for 5 × 10^5^ cells but calculated to 10^6^ cells for comparison (for the original measured values of LNCaP see SI, Figure S18).

To compare cell uptake with the reference cell lines PC-3 and LNCaP, a separate single-time-point (2 h) internalization assay was performed. [^111^In]In-RM2 was internalized into CHO-K1-GRPR cells up to 5.7% ± 0.4% AD/10^6^ cells and showed a membrane-bound amount of 37.8% ± 2.9% AD/10^6^ cells (*n* = 3, Figure 5A). For PC-3 cells, a value of 8.1% ± 0.6% AD/10^6^ cells was obtained for the internalized fraction and 37.7% ± 4.3% AD/10^6^ cells for the membrane-bound fraction (*n* = 3, Figure 5A).

[^99m^Tc]Tc-PSMA-I&S showed an internalization (not corrected for non-specific binding) of 28.2% ± 1.2% AD/10^6^ cells in CHO-K1-PSMA cells (*n* = 3, Figure 5B) and reached a value of 29.4% ± 3.6% AD/10^6^ cells for LNCaP cells (*n* = 3, Figure 5B). Membrane-bound [^99m^Tc]Tc-PSMA-I&S was found to be 20.1% ± 2.5% AD/10^6^ cells for CHO-K1-PSMA cells (*n* = 3, Figure 5B) and 17.0% ± 1.8% AD/10^6^ cells for LNCaP cells (*n* = 3, Figure 5B).

Validation studies and identification of potential mutual interferences between [^111^In]In-RM2 and [^99m^Tc]Tc-PSMA-I&S on their targets were carried out by measuring their ability to compete with unlabeled PSMA-I&S or RM2, respectively. The experimental setup was analogous to the internalization assay described above. The membrane-bound amount of [^111^In]In-RM2 alone reached a value of 36.6% ± 2.9% AD/10^6^ cells and in the presence of PSMA-I&S still reached 35.8% ± 3.5% AD/10^6^ cells (*n* = 3, Figure 5A, green column). With regard to the internalized fraction, [^111^In]In-RM2 alone exhibited 5.7% ± 0.2% AD/10^6^ cells and 5.1% ± 0.5% AD/10^6^ cells in the presence of PSMA-I&S (*n* = 3, Figure 5A, orange column).

[^99m^Tc]Tc-PSMA-I&S alone displayed a value of 15.7% ± 2.9% AD/10^6^ cells for the membrane-bound fraction and 17.3% ± 3.2% AD/10^6^ cells in the presence of RM2 (*n* = 3, Figure 5B, green column). The internalized fraction revealed 24.1% ± 5.4% AD/10^6^ cells for [^99m^Tc]Tc-PSMA-I&S alone and gave a value of 26.9% ± 5.7% AD/10^6^ cells when co-incubated with RM2 (*n* = 3, Figure 5B, orange column). Hence, cellular receptor/enzyme-mediated uptake of [^111^In]In-RM2 and [^99m^Tc]Tc-PSMA-I&S did not change in the presence of the unlabeled PSMA-I&S or RM2, respectively.

Non-transduced CHO-K1 cells were used as a negative control. Both [^111^In]In-RM2 and [^99m^Tc]Tc-PSMA-I&S showed negligible binding to CHO-K1 cells (*n* = 3 and *n* = 2 in triplicate, Figure 5).

Specific binding of [^111^In]In-RM2 and [^99m^Tc]Tc-PSMA-I&S to their respective target structures (i.e., GRPR and PSMA) was verified by co-incubation with a 1000-fold excess of BBN and a 1000-fold excess of 2-PMPA (‘Blocking’ condition), respectively. Thereby, the amount of activity taken up by the transduced cells could be notably reduced and only non-specific binding remained.

**Figure 6:**
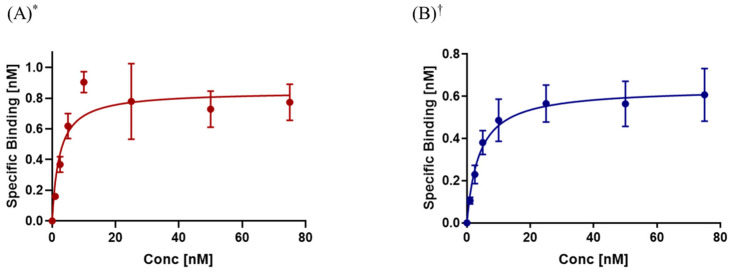
Saturation experiments with radiolabeled peptides (**A**) [^111^In]In-RM2 and (**B**) [^99m^Tc]Tc-PSMA-I&S using CHO-K1-GRPR or CHO-K1-PSMA cells, respectively. K_d_ and B_max_ values were determined by non-linear regression using GraphPad Prism 8. Data are depicted in mean ± SD (* *n* = 3 and ^†^ *n* = 4 in triplicate) and normalized to 10^6^ cells per well.

Saturation binding experiments were carried out with increasing concentrations of the radiolabeled peptides for 2 h at 4 °C. The K_d_ value for [^111^In]In-RM2 on CHO-K1-GRPR cells was found to be 2.33 ± 0.40 nM while the B_max_ was 0.84 ± 0.03 nM (Figure 6A, Table 1). This B_max_ value corresponds to 5.06 × 10^5^ binding sites per cell. The K_d_ determination of [^99m^Tc]Tc-PSMA-I&S using CHO-K1-PSMA cells revealed 3.84 ± 0.44 nM, and for the B_max_, 0.64 ± 0.02 nM was obtained (Figure 6B, Table 1). This B_max_ value represents 3.85 × 10^5^ binding sites per cell (binding sites per cell were calculated according to the equations in Section 3.4.1 in the SI).

### 2.4. Dual-Isotope SPECT Phantom Measurements

**Figure 7:**
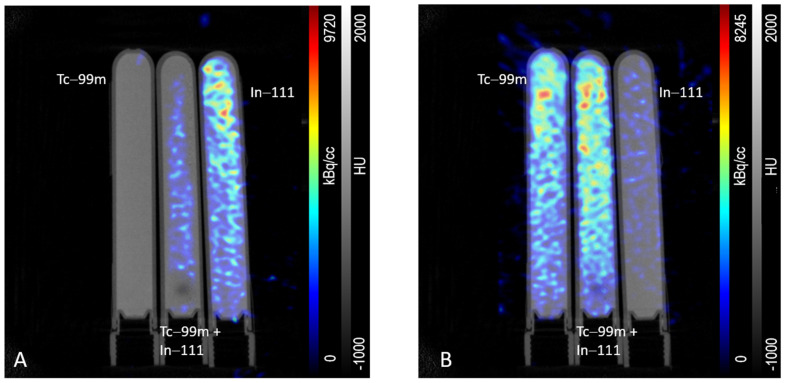
Tubes containing 12.11 MBq ^99m^Tc (left), 11.79 MBq ^99m^Tc and 11.34 MBq ^111^In (middle), and 10.20 MBq ^111^In (right) were acquired and reconstructed using either (**A**) the ^111^In energy window (154–188 keV) or (**B**) the ^99m^Tc energy window (126–154 keV).

To set up the imaging protocol, three tubes were filled with (1) 12.11 MBq ^99m^Tc, (2) 11.79 MBq ^99m^Tc and 11.34 MBq ^111^In and (3) 10.20 MBq ^111^In and placed in the field of view of the preclinical imaging scanner. SPECT data were acquired for 10 min using an energy window of 154–188 keV (^111^In frame) or 126–154 keV (^99m^Tc frame) following a computed tomography (CT) acquisition as reference and for attenuation/scatter correction. After reconstruction, almost no crosstalk of ^99m^Tc into the ^111^In window could be detected (Figure 7A, very left tube), and only minor crosstalk of ^111^In 171 keV photons into the ^99m^Tc window could be observed (Figure 7B, very right tube).

### 2.5. In/Ex Vivo Evaluation

#### 2.5.1. *µ*SPECT/CT Imaging

**Figure 8:**
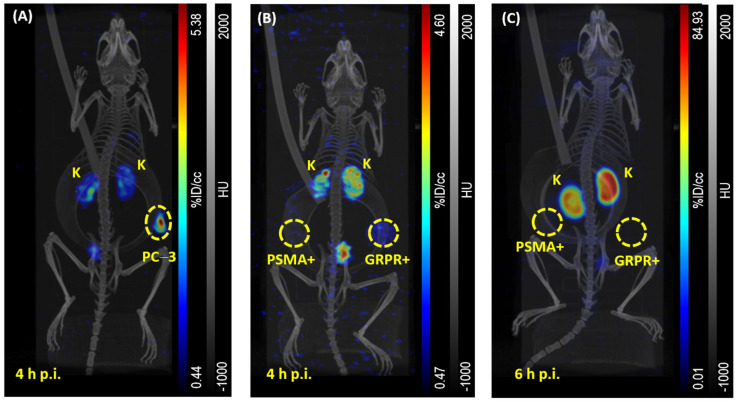
Representative maximum intensity projections of *µ*SPECT/CT images (**A**) 4 h p.i. of [^111^In]In-RM2 (PC-3 xenograft, dorsal right flank), (**B**) 4 h p.i. of [^111^In]In-RM2 (CHO-K1-GRPR xenograft, dorsal right flank and CHO-K1-PSMA xenograft, dorsal left flank) and (**C**) 6 h p.i. of [^99m^Tc]Tc-PSMA-I&S (CHO-K1-GRPR xenograft, dorsal right flank and CHO-K1-PSMA xenograft, dorsal left flank). Tumor xenografts are highlighted by yellow dashed circles. *PSMA+* refers to CHO-K1-PSMA xenografts, *GRPR+* refers to CHO-K1-GRPR xenografts; *K* = Kidneys. Data of subsequent biodistribution of (**A**) and (**B**) correspond to mouse #3 and mouse #6 in Figure 9, respectively. In Figure 10, data of subsequent biodistribution of (**C**) are displayed as *Single Tracer, Dual Xenograft, 6.6 h p.i., 0.67 nmol [#13]*.

To determine the optimal time point and injected dose for subsequent DI imaging, single-tracer SPECT imaging was performed for up to 25 h p.i. using different injected activities (and hence amounts of substances). In all imaging studies, no notable tracer uptake in CHO-K1-GRPR and CHO-K1-PSMA xenografts could be detected (Figure 8B,C), despite fast tumor growth (1.5–2 weeks) and a final tumor volume of 100–800 mm^3^. In contrast, [^111^In]In-RM2 clearly demonstrated tumor uptake using the PC-3 xenograft model (Figure 8A).

**Figure 9:**
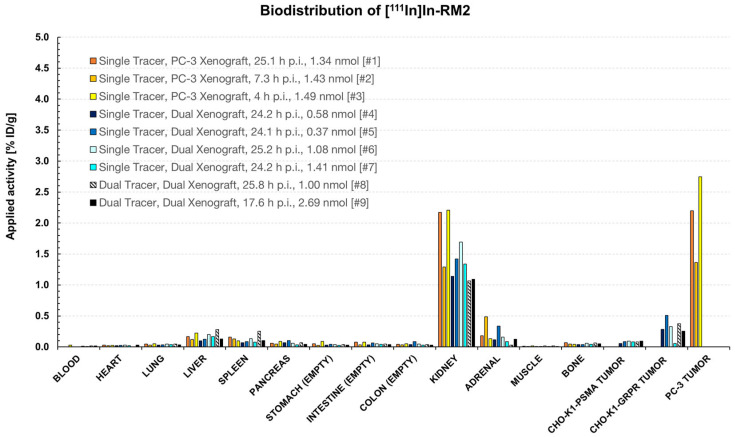
Biodistribution data (% ID/g) of [^111^In]In-RM2 in female NOD SCID mice at different time points, with different amounts of substance and different activities injected (5.22–34.5 MBq). Mice were sacrificed after *µ*SPECT/CT studies in which the ideal imaging time point after tracer administration was planned to be identified. Due to the different test parameters, it was not possible to group data according to biodistribution time, amount of substance, applied activity, etc. Single tracer: only [^111^]In-RM2 was injected; Dual tracer: [^111^In]In-RM2 and [^99m^Tc]Tc-PSMA-I&S were injected; PC-3 Xenograft: Mice bearing a single PC-3 xenograft. Dual Xenograft: Mice bearing CHO-K1-GRPR and -PSMA xenografts; Animal identification numbers are given in square brackets and the exact values used for this bar diagram are given in the SI (Table S4, Section 3.6.1.2).

**Figure 10:**
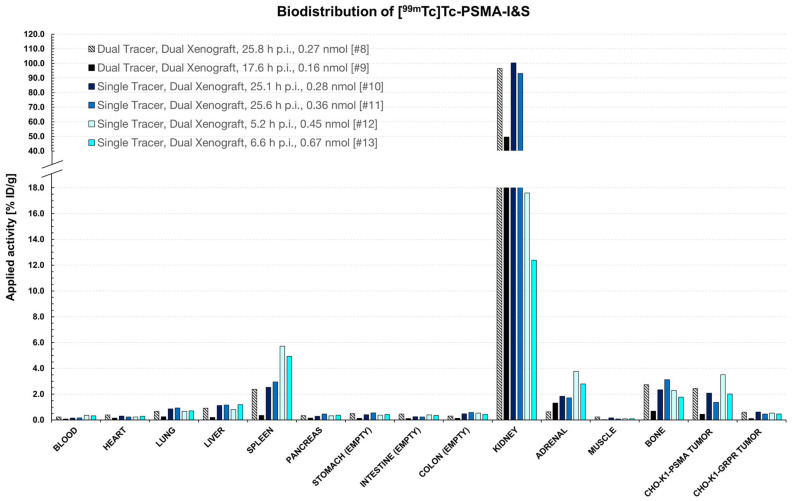
Biodistribution data (% ID/g) of [^99m^Tc]Tc-PSMA-I&S in female NOD SCID mice at different time points, with different amounts of substance and different activities injected (6.00–54.0 MBq). Mice were sacrificed after *µ*SPECT/CT studies in which the ideal imaging time point after tracer administration was planned to be identified. Due to the different test parameters, it was not possible to group data according to biodistribution time, amount of substance, applied activity, etc. Single tracer: only [^99m^Tc]Tc-PSMA-I&S was injected; Dual tracer: [^99m^Tc]Tc-PSMA-I&S and [^111^In]In-RM2 were injected; Dual Xenograft: Mice bearing CHO-K1-PSMA and -GRPR xenografts. Animal identification numbers are given in square brackets, and the exact values used for this bar diagram are given in the SI (Table S5, Section 3.6.1.2).

Mice with single xenografts were only used for single-tracer ([^111^In]In-RM2) reference studies with PC-3 cells. Dual xenograft mice (CHO-K1-PSMA + CHO-K1-GRPR cells injected into different flanks) were used for single and dual-tracer studies ([^111^In]In-RM2+ [^99m^Tc]Tc-PSMA-I&S).

#### 2.5.2. Biodistribution Studies

Biodistribution studies were conducted subsequently to *µ*SPECT/CT experiments for validation. The results are summarized in Figure 9 and Figure 10.

Notable tracer uptake could only be detected in PC-3 xenografts (1.36–2.75% ID/g, 4-25.1 h p.i.). CHO-K1-GRPR xenografts (GRPR^+^) showed slightly higher [^111^In]In-RM2 uptake (0.06% ID/g, 24.2 h p.i.–0.51% ID/g, 24.1 h p.i.) than CHO-K1-PSMA (GRPR^-^) xenografts (0.05% ID/g, 24.2 h p.i.–0.09% ID/g, 17.6 and 25.2 h p.i.). However, these values were in the range of other non-target organs (e.g., liver or spleen).

CHO-K1-PSMA xenografts (PSMA^+^) showed slightly higher [^99m^Tc]Tc-PSMA-I&S uptake (0.44% ID/g, 17.6 h p.i.–3.51% ID/g, 5.2 h p.i.) than CHO-K1-GRPR (PSMA-) xenografts (0.12% ID/g, 17.6 h p.i.–0.61% ID/g, 25.1 h p.i.). However, also for this radioligand, the values were in the range of other non-target organs (e.g., adrenal or bone).

#### 2.5.3. Western Blot Analysis of Cell and Tissue Lysates

**Figure 11:**
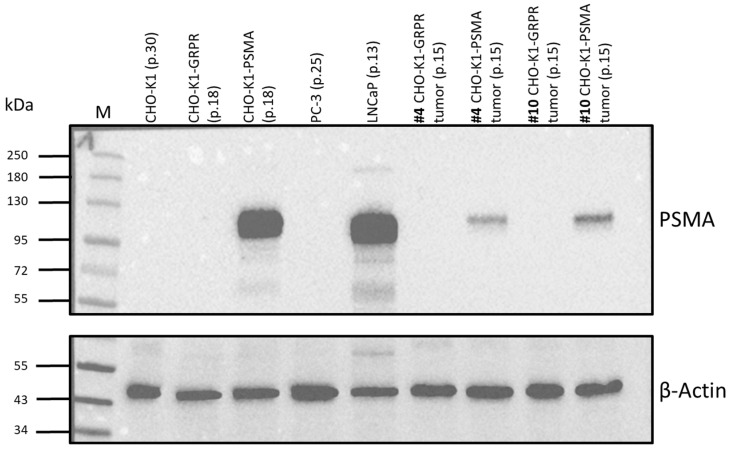
Representative Western blot for PSMA expression (**above**) of various cell (CHO-K1, CHO-K1-GRPR, CHO-K1-PSMA, PC-3, and LNCaP) and tissue (CHO-K1-GRPR + CHO-K1-PSMA dual xenograft) lysates. Mice for tissue lysates were injected with [^111^In]In-RM2 (#4) or [^99m^Tc]Tc-PSMA-I&S (#10) and tissue lysates prepared after radionuclide decay. Passage numbers in parentheses indicate the passages for which cell lysates were prepared or, for tissue lysates, the passage number at the time point of inoculation in mice. β-Actin (**below**) was used as loading control. M: Color Prestained Protein Standard, Broad Range (10–250 kDa, New England Biolabs).

As depicted in Figure 11, only CHO-K1-PSMA and LNCaP cell lysates revealed the expected PSMA bands at approx. 100 kDa (columns 4 and 6 from left). Tissue lysates of GRPR^+^ tumors showed no bands (columns 7 and 9 from left), but also CHO-K1-PSMA tumor lysates displayed PSMA bands with notably reduced intensity (columns 8 and 10 from left).

## 3. Discussion

The simultaneous application of two (or more) single tracers targeting different cell-surface-exposed proteins in a DI approach has high potential from a clinical perspective [14,27]. We hypothesize that the application of several tumor-specific ligands at the same time in a multi-radiotracer approach could be used to improve the management of the disease by better identification of lesions as well as understanding their molecular biology. For a proof-of-concept study, the two tumor-specific membrane proteins PSMA and GRPR were selected because they are well-known targets for clinical imaging of PCa. We assume a potential benefit for the patient by combining ligands for these specific targets, as they could serve as valuable tools to investigate spatial and temporal tumor heterogeneity [14]. The respective ligands for these targets, PSMA-I&S and RM2, were synthesized and labeled with different SPECT radionuclides. More precisely, PSMA-I&S was radiolabeled with ^99m^Tc and RM2 with ^111^In. The different main γ-energies of the radioisotopes (^99m^Tc = 140.5 keV, ^111^In = 171 keV, and 245 keV [28]) allow for discrimination of the radionuclides, and thus also for discrimination of the radiolabeled compounds in vivo by SPECT [29,30]. Preceding measurements of phantoms containing ^99m^Tc, ^111^In, or a mixture of both nuclides verified distinguishability by the in-house Inveon *µ*SPECT/CT scanner system with only minor crosstalk of ^111^In onto the ^99m^Tc energy window (126–154 keV) and almost no crosstalk of ^99m^Tc onto the ^111^In energy window (154–188 keV) (Figure 7).

Identifying an appropriate preclinical in vitro and in vivo model for this study was challenging but essential. In the past years, many cell lines have been developed in order to address several aspects of human PCa [31]. To date, the most widely used cell lines in preclinical PCa studies are GRPR-positive PC-3 (doubling time ~33 h) and PSMA-positive LNCaP (doubling time ~60 h) cells, exhibiting varying growth rates, as well as DU145 (doubling time ~34 h) [31,32]. However, different tumor growth rates adversely affect the generation of a mouse model bearing two xenografts of different cell line origin. When inoculated into the same animal, one tumor usually has a growth advantage. As a result, the second tumor either does not grow at all or remains very small [32,33].

Recently, a bispecific PSMA-617/RM2 heterodimer targeting PSMA and GRPR was investigated using two different animal models (subcutaneous PC-3 or LNCaP xenografts) by Liolios C et al. [34]. Radio-iodinated bispecific heterodimers were successfully evaluated in vivo using PC-3-PIP (GRPR and PSMA-positive) xenografts [35]. However, a reliable animal model allowing targeting both biomarkers in the same subject but within different lesions would more accurately reflect tumor heterogeneity in patients. In order to realize an optimized in vitro and in vivo model for preclinical dual-tracer SPECT imaging, we aimed at establishing two different xenografts with the same doubling time and target expression density in the same animal. Therefore, we focused on the use of transduced CHO-K1 cells stably expressing the human GRPR or PSMA protein as an alternative to the commonly used PC-3 or LNCaP cell lines. CHO-K1 cells underwent lentiviral transduction for GRPR or PSMA overexpression, as this method is considered a powerful tool to enable stable transgene/target expression [36]. Overexpression of human PSMA was determined by Western blot analysis for one single clone, i.e., clone 5, which was further used in this study. Expression of human GRPR was assessed and confirmed for several clones by RT-qPCR, as no human-specific antibodies for Western blot analysis were available, and the receptor protein can be present in different glycosylated variants, which impairs the detection of specific GRPR isoforms [37].

Both newly generated cell lines, CHO-K1-GRPR and CHO-K1-PSMA, were investigated by incubation with the well-established radioligands [^111^In]In-RM2 and [^99m^Tc]Tc-PSMA-I&S. Saturation experiments for both cell lines revealed a similar number of binding sites (5.06 × 10^5^ binding sites/cell for CHO-K1-GRPR and 3.85 × 10^5^ binding sites/cell for CHO-K1-PSMA, Table 1). Receptor saturation values of [^111^In]In-RM2 with CHO-K1-GRPR are similar to the literature data (5.5 × 10^5^ binding sites/cell), even though PC-3 cells were used [38]. For [^99m^Tc]Tc-PSMA-I&S, a direct comparison was not possible due to a lack of data for B_max_. However, across some of the reported literature on different urea-based PSMA inhibitors [39,40], B_max_ values were in the range of 0.27 to 0.73 nM. The B_max_ found for [^99m^Tc]Tc-PSMA-I&S in our study (0.64 ± 0.02 nM, CHO-K1-PSMA cells) is therefore within the range of these literature data (obtained for LNCaP cells).

Since the number of binding sites for both cell lines, CHO-K1-GRPR and CHO-K1-PSMA, were in the same order of magnitude, a similar receptor/enzyme occupation was to be expected, provided that both radioligands are injected in equal amounts. Yet, other factors could still influence the tumor uptake (i.e., differences in apparent molar activity and different clearance kinetics of the radiolabeled compounds as well as varying detection efficiencies of the *µ*SPECT device for the respective nuclides). However, differences in tracer uptake caused by a notable discrepancy in target expression were not expected. CHO-K1 cells did not show a noticeable change in the growth rate after transduction with *GRPR* or *PSMA*, and both transduced cell lines exhibited a very similar doubling time of ~24 h [41] throughout the entire in vitro experiments. Hence, similar growth rates for both tumor xenografts in mice were anticipated and could be confirmed by reproducible development of dual xenografts within the same animal 1.5–2 weeks after inoculation.

In a single-time point (2 h) internalization assay, CHO-K1-GRPR and PC-3 revealed a comparable cellular uptake of [^111^In]In-RM2 (internalized and membrane-bound fraction, Figure 5A). Thus, CHO-K1-GRPR (doubling time ~24 h) was assumed to be a reliable option as an alternative GRPR-overexpressing cell line instead of PC-3 (doubling time ~33 h). Likewise, internalized and membrane-bound fractions of [^99m^Tc]Tc-PSMA-I&S reached very similar values in CHO-K1-PSMA and LNCaP cells (Figure 5B). Considering the slow cell proliferation of LNCaP (doubling time ~60 h), CHO-K1-PSMA (doubling time ~24 h) was considered as a more favorable option as a PSMA-overexpressing cell line in the following *in*/ex vivo experiments.

In addition, blocking experiments with RM2 and 2-PMPA confirmed GRPR and PSMA-specific cellular binding and uptake of the investigated radiotracers. Control internalization experiments with [^111^In]In-RM2 and [^99m^Tc]Tc-PSMA-I&S in the presence of excess unlabeled PSMA-I&S and RM2, respectively, revealed that tracer uptake is not affected in the presence of the other ligand. We thus expected that in animal studies, the tracer uptake should not be impaired in the presence of the other radioligand either.

For in vivo experiments, approximately the same passage (±1 passage number) of both cell lines was used for inoculation to ensure similar cell viability, target incidence, and density. Moreover, tracer uptake (and thus target expression) was confirmed three days prior to inoculation in cell uptake experiments. Based on the in vitro results of both transduced cell lines and the results of the DI SPECT phantom measurements (Figure 7), xenografts were presumed to be clearly visible, well distinguishable, and target expression could be precisely assigned both qualitatively and quantitatively.

However, no notable tracer uptake in CHO-K1-GRPR and CHO-K1-PSMA xenografts could be detected, despite fast tumor growth (1.5–2 weeks) and a final tumor volume of 100–800 mm^3^. As [^111^In]In-RM2 and [^99m^Tc]Tc-PSMA-I&S show different pharmacokinetics, it was crucial to determine an optimal time point for imaging of both tracers. Therefore, single tracers were initially administered in order to generate reference data (Figure 8, Figure 9 and Figure 10). No activity uptake in both tumor xenografts at various imaging time points could be detected, wherefore the images of dual-tracer animals are not shown, but only images of single-tracer animals. This allows for a better comparison with the reference single-tracer animal in which only [^111^In]In-RM2 was administered to a PC-3 single xenograft animal (Figure 8).

The biodistribution data (Figure 9 and Figure 10) confirmed low tracer uptake in CHO-K1-GRPR/-PSMA xenografts with no notable difference between single and dual-tracer animals (0.06–0.51% ID/g for [^111^In]In-RM2 in single-tracer mice #4–#7 vs. 0.26–0.38% ID/g for [^111^In]In-RM2 in dual-tracer mice #8 and #9 and 1.37–3.51% ID/g for [^99m^Tc]Tc-PSMA-I&S in single-tracer mice #10–#13 vs. 0.44–2.45% ID/g for [^99m^Tc]Tc-PSMA-I&S in dual-tracer mice #8 and #9). Only the PC-3 xenografts clearly displayed elevated [^111^In]In-RM2 uptake (1.36% ID/g–2.75% ID/g, Figure 8 and Figure 9).

For comparison, the literature data of Mansi et al. [37] revealed an [^111^In]In-RM2 uptake of 6.84 ± 1.02% ID/g at 24 h p.i. (*n* = 4) in PC-3 xenografts. Despite certain differences in experimental setup (3-week-old female nude mice, i.v. injection of 10 pmol of radiolabeled peptides, about 0.18 MBq, 100 μL, *n* = 4 in Mansi et al. vs. 6–8-week-old female NOD SCID mice, i.v. injection of 1.34–1.49 nmol of radiolabeled peptides, 31.6–33.6 MBq, 100–150 μL, *n* = 3 for PC-3 in this study), it is evident that both xenografts based on transduced cell lines show only minor [^111^In]In-RM2 uptake compared to xenografts based on the established PC-3 cells.

For [^99m^Tc]Tc-PSMA-I&S, no comparative experiments in male LNCaP-bearing mice could be performed. The respective animal ethics only allowed for the use of female mice in which androgen-dependent LNCaP xenografts cannot grow due to the absence of male androgens [42]. According to previously published data [43], LNCaP-based xenografts revealed a [^99m^Tc]Tc-PSMA-I&S uptake of 8.28 ± 3.27% ID/g at 1 h p.i. (6–8-week-old male CB-17 SCID mice, i.v. injection of 0.1 nmol of radiolabeled peptides, about 3–4 MBq, *n* = 3–5). In our experiments, no mice were dissected at this early time point, so a true comparative analysis cannot be included here.

Clearly detectable [^111^In]In-RM2 uptake in PC-3 xenografts in the reference experiment (positive control, Figure 8A and Figure 9) indicated a loss of receptor/enzyme expression in vivo in xenografts derived from both transduced cell lines. This assumption could be supported by Western blot studies in which some of the dissected CHO-K1-PSMA xenografts were analyzed in comparison to cell lysates exhibiting approximately the same passage numbers, which were later used for inoculation in mice and hence for xenograft formation (Figure 11). CHO-K1-PSMA tumor lysates displayed PSMA bands with notably reduced intensity (Figure 11, columns 8 and 10 from left) when compared to the respective cell lysates. A reduced band intensity for tissue lysates in comparison to cell lysates might be expected on one hand due to the fact that for tissue lysates, the CHO-K1-PSMA cell density might be lower in the sample. Despite the same protein load (15 µg per pocket), other types of cells and hence other proteins will be more present in tissue lysates than in cell lysates, in which generally only one type of cell is present. On the other hand, considering the rather low [^99m^Tc]Tc-PSMA-I&S uptake in CHO-K1-PSMA cells in biodistribution studies (0.44–3.51% ID/g), it can be assumed that reduced enzyme expression is the reason for such low tracer uptake and reduced band intensity in Western blots.

An exclusively human-specific primary anti-GRPR antibody was not available. The mouse monoclonal anti-GRPR antibodies we used (D-1, sc-398549 SCB, Santa Cruz Biotechnology, Dallas, TX, USA, or ABR-002, Alomone Labs, Ltd., Jerusalem, Israel) were also able to bind to hamster GRPR in non-transduced CHO-K1 and CHO-K1-PSMA cells. In addition, it was very difficult to detect specific GRPR isoforms due to the large number of differently glycosylated variants of this receptor [37]. As a consequence, the blots were not interpretable concerning a potential loss of GRPR expression in vivo.

Selection antibiotics were absent in in vivo studies but present throughout all in vitro experiments, which could have led to this huge discrepancy in uptake for both radioligands, [^111^In]In-RM2 and [^99m^Tc]Tc-PSMA-I&S. Similar observations (poor conservation of biomarker expression) have been made for human A_3_ adenosine receptor-expressing HT-29 cells [44]. However, mechanistic studies on this reduced target expression in vivo were not included. Loss of biomarker expression has not been reported for PC-3-PIP, a GRPR- and PSMA-positive cell line that was generated by transduction of PC-3 cells using a VSV-G pseudotyped lentiviral vector system [45]. Likewise, stable biomarker expression, which could also be preserved in vivo, was observed for PC-3 PIP-luc cells, which were transduced with RediFect Red-FLuc-GFP lentiviral particles to stably express GRPR, PSMA, and GFP-luciferase [46]. This emphasizes that despite positive in vitro results, a new concept like dual-tracer imaging cannot necessarily be evaluated by using new methods (i.e., transduced cell lines). Ideally, the methods planned to be used for new concepts should be validated first, also in vivo.

We propose that by using alternative PCa-associated in vivo models (e.g., LNCaP and PC-3 instead of transduced cell lines), a combination of [^99m^Tc]Tc-PSMA-I&S and [^111^In]In-RM2 could lead to a positive outcome of the study. Different doubling times of the cell lines and hence non-uniform tumor growth, however, would require a more elaborate timeline for dual xenograft generation (cells need to be inoculated several days apart) and optimized scoring protocols. The use of another cell line than CHO-K1 as a basis for transduction might also be thinkable. This approach, however, bears again the risk of in vivo loss of biomarker, and additional animals would be needed to verify sustained target expression in vivo. Due to this negative animal ethical aspect and also due to the comparatively time-efficient and expedient strategy to use cells, which are known to preserve their PSMA/GRPR expression in vivo, the use of LNCaP and PC-3 cells is assumed to be the more expedient and successful strategy. Thereby, at least a proper ligand uptake in tumor lesions can be expected.

For future dual-tracer applications and due to the technical constraints also described in Section 4.4 (the function of list-mode acquisition could not be implemented at the Siemens Inveon^®^ device, instead sequential scans shortly after each other were performed, considered as quasi-simultaneous), several assumptions were made for the sake of simplicity and feasibility, although not leading to absolute quantitatively correct results. At the imaging time points (1–24 h for [^99m^Tc]Tc-PSMA-I&S and [^111^In]In-RM2), the activity in the animal (t_1/2_ ≥ 6 h for both tracers) was considered as virtually stable during two consecutive scans (at least for 10–20 min SPECT scans). For early imaging time points (e.g., 0–30 min p.i.) and radioisotopes with short half-life (e.g., ^11^C) or rapidly metabolized probes, these assumptions cannot be made. Moreover, for certain single or dual-tracer animals, the SPECT imaging period was increased up to 60 min (Section 4.5.1) as we aimed at an improved image quality due to very low tracer uptake in the lesions. For future experiments, this imaging period needs to be considerably reduced, since two consecutive 60 min SPECT scans cannot be considered as quasi-simultaneous. In any of these cases, the use of an alternative scanner system that is capable of acquiring list-mode data, which can be reconstructed afterwards by choosing the corresponding energy window, would be more expedient. This would allow for true simultaneous dual-tracer SPECT imaging as it could be applied in the clinic. If not available, certain compromises, as mentioned above, need to be taken into consideration at least for preclinical dual-tracer SPECT studies.

Based on this proof-of-principle study, we identified several scientific and technical aspects that need to be considered for dual-tracer approaches already in the planning phase. First, it is necessary to identify and ideally exclude potential competition of the radiotracers. For this purpose, suitable in vitro studies need to be set up or at least adjusted as it was performed in this study to check for potential competitive effects of the used compounds, PSMA-I&S and RM2, on each other. Thus, mutual blocking effects of the radiotracers in vivo could be excluded, preserving signal selectivity for each individual target.

Second, due to different pharmacokinetics of the radiotracers, and hence different time points for each optimal tumor-to-background ratio, several time points need to be chosen for imaging in order to identify the optimal tumor-to-background ratio of the dual-tracer cocktail. In case of simultaneous biomarker injection, it is more likely to identify a reasonable compromise for the best imaging time point with ligands exhibiting a similar excretion duration than for ligands with strongly varying clearance rates from non-target tissue. However, due to absent activity uptake in tumor xenografts, no optimal imaging time point could be identified for the dual tracer. For the same reason, the *µ*SPECT/CT scans were not analyzed quantitatively but only qualitatively.

Third, dual-tracer scans require specific injection protocols for dual-tracer administrations. For simultaneous injections, as conducted in this study, both compounds are mixed and injected via the same catheter. For preclinical studies in mice, this requires that the overall maximum i.v. injection volume of 200 µL is not exceeded and that the molar activities of the individual single tracers are optimized or increased accordingly in advance (Section 4.1). Besides, due to co-incubation for a certain period, stability of the individual tracers within the dual-tracer mixture needs to be ensured and hence, quality control procedures for the radiotracers need to be adjusted. Simultaneous injections could facilitate biodistribution analysis (same biodistribution period for both ligands) and could be advantageous for dynamic imaging studies. However, the presence of both nuclides in the catheter used for i.v. injection leads to a very difficult procedure to calculate back to the initially applied activity (time-consuming and cumbersome γ-counter measuring procedure as described for dual-isotope probes in Section 4.3 and Section 3.6.1.1 in the SI). By using two separate catheters, the applied activity for both tracers can be calculated as simply as for single-tracer administrations, but a true simultaneous application will not be possible by this approach. This, however, is not mandatory if static images at specific time points are acquired, and it might be advantageous in case the clearance kinetics of both tracers are too different. In certain cases, it might be even better to choose a sequential application protocol and apply the second tracer, e.g., 24 h after the first one, to reach optimal tumor-to-background ratios for both tracers. In preclinical settings, however, the maximum intravenously injected volume of a sequential administration does not differ from that of a simultaneous injection (200 µL) if both injections are conducted within 24 h. It might still be advisable to conduct adjusted quality control procedures (i.e., co-incubation studies) of the radiotracers if the compounds are to be injected in quick succession. In the event of a catheter obstruction, both tracers can then be safely applied via one catheter.

A simultaneous injection or injections temporally very close to each other also require that both tracers can be synthesized more or less simultaneously or at least made available at the same time with a sufficiently high radiochemical purity (RCP ≥ 95%). This needs to be considered upfront in synthesis logistics (i.e., availability of isotopes, equipment, human resources, etc.).

Eventually, appropriate analysis methods to correctly quantify two different radionuclides in one sample are required. Biodistribution analysis requires specific calculation methods in case of insufficient distinguishability of the nuclides by the used equipment, as conducted in this study (Section 4.3 and Section 3.6 in the SI). Based on data acquired over a prolonged measuring period (2–3 weeks), activities of the probes at the start of the measurement can be calculated specifically for each individual isotope and hence allow for a quantitative analysis. However, in the case of more routine use, this γ-counter set-up requires certain optimizations for a more accurate calculation of the individual isotope contents, in particular for probes with low activity in general (70.6–79.7% accuracy for ^111^In containing only 5 kBq in total of the isotope mixture and 68.5–81.3% accuracy for ^99m^Tc in probes containing ≤ 5% ^99m^Tc, Section 3.5, SI). Alternatively, measurements with a calibrated γ-spectrometer could be envisaged. However, such an instrument was not available for this study. Therefore, we want to present a valuable and reliable method for quantitative analysis of dual-isotope probes that is not based on γ-spectrometry and thus could also be used for similar projects in which no calibrated γ-spectrometer is available. For the dual-tracer *µ*SPECT/CT scans, it should be a matter of course to conduct phantom measurements at the respective scanner system to set up acquisition and reconstruction protocols with suitable energy windows that allow for quantification of the different radioisotopes after a single measurement.

Compared to classical single-tracer studies, these scientific and technical challenges need to be taken into account. With our strategies to address many of these points, we aim to pave the way towards future, optimized dual-tracer SPECT applications.

## 4. Materials and Methods

### 4.1. Syntheses

Syntheses of precursor peptides RM2 and PSMA-I&S, as well as of the cold standard ^nat^In-RM2, were conducted according to previously published procedures [38,43,47,48] with some minor modifications. The exact procedures and further details on instrumentation and chemicals are given in the Supporting Information.

*Preparation of [^111^In]In-RM2*. 5–10 µL of NaOAc (in H_2_O, 0.3 M) were added to 50 µL of [^111^In]InCl_3_ (in 0.02 M HCl, 370 MBq/mL, pH = 1.5) in order to reach a pH of 4–4.5. Subsequently, 5 µL of RM2 stock solution (in H_2_O, 1.00 mM, 5.00 mmol) were added to the mixture and heated for 10–20 min at 95 °C. [^111^In]In-RM2 was obtained in a radiochemical purity (RCP) of >95% (*n* > 10) as determined by radio-RP-HPLC (R_t_ = 5.8 min, 85–60% A in 15 min, 3 mL/min, λ = 220 nm; SI, Figure S15).

For in vivo studies, an optimized labeling procedure was established to obtain [^111^In]In-RM2 in higher apparent molar activities (A_m_). In a 0.5 mL Protein LoBind^®^ tube (Eppendorf, Cat. Nr. 0030108094), 8 µL of NaOAc*3 H_2_O (in H_2_O, 0.3 M) were added to 50 µL of [^111^In]InCl_3_ to reach a pH of 4–4.5. Subsequently, 2 µL of RM2 stock solution (in H_2_O, 1.00 mM, 2.00 nmol) was added and heated for 10 min at 95 °C. The solution was mixed (after 5 min) and placed back into the heating block. Thus, apparent A_m_ of 13.7–23.1 MBq/nmol (*n* = 5) could be reached with RCPs ≥ 95%. The reaction was also conducted successfully with twice the amount of reactants (i.e., 100 µL [^111^In]InCl_3_, 16 µL 0.3 M NaOAc*3 H_2_O, 4 µL-RM2 stock solution) and heating it for 10 min at 95 °C. The resulting apparent A_m_ ranged from 18.8 to 24.7 MBq/nmol (*n* = 9) with RCPs ≥ 95%.

*Preparation of [^99m^Tc]Tc-PSMA-I&S*. [^99m^Tc]TcO_4_^−^ for radiosyntheses was obtained from a ^99^Mo/^99m^Tc generator by elution with 0.9% NaCl. Radiolabeling was performed in a kit-based radiolabeling procedure. The lyophilized kits were prepared in analogy to a previously published procedure [30], with minor modifications (a detailed procedure can be found in the SI). For radiolabeling, 200 µL of [^99m^Tc]TcO_4_^−^ (~150–250 MBq) in 0.9% NaCl were added to the kit (pH = 7–7.5), containing 5 nmol of PSMA-I&S. In total, 0.7 µL NaOH (1 M) were added to reach a pH = 8–8.5 of the final reaction mixture. The solution was heated to 90 °C for 20 min. [^99m^Tc]Tc-PSMA-I&S was obtained in a RCP of >95% (*n* > 10) as determined by radio-RP-HPLC (linear gradient from 85 to 60% A in 15 min with a flow rate of 3 mL/min (λ = 220 nm, R_t_ = 4.8 min; SI, Figure S16).

For in vivo studies, an optimized labeling procedure was established to obtain [^99m^Tc]Tc-PSMA-I&S in higher A_m_. Instead of 5 nmol kits, kits containing 2 nmol of PSMA-I&S were used (detailed procedure on 2 nmol kit preparation can be found in the SI). All other reaction steps (addition of [^99m^Tc]TcO_4_^−^, pH adjustment, and heating) were conducted as described above. [^99m^Tc]Tc-PSMA-I&S was obtained in a RCP of >95% (*n* = 9) as determined by radio-RP-HPLC.

### 4.2. Cell Culture and In Vitro Evaluation

All cell culture reagents were purchased from Thermo Fisher Scientific (Vienna, Austria) or Sigma Aldrich (Vienna, Austria) unless stated otherwise. PC-3 and LNCaP cells were purchased from ATCC and cultivated in RPMI-1640 medium supplemented with 10% heat-inactivated fetal bovine serum (FBS), 2 mM L-glutamine, 100 units/mL penicillin, and 100 μg/mL streptomycin. Chinese hamster ovary cells (CHO-K1) were obtained from ATCC and cultivated in Gibco^TM^ Ham’s F-12 Nutrient Mix medium supplemented with 10% heat-inactivated FBS, 100 units/mL penicillin, and 100 μg/mL streptomycin. CHO-K1 cells transduced with human GRPR or PSMA (CHO-K1-GRPR or CHO-K1-PSMA) were cultivated with the addition of 2.5 μg/mL puromycin. All cell lines were kept in culture under a humidified atmosphere (37 °C, 5% CO_2_) and used for in vitro or in vivo experiments when 80% confluence was reached. Cells were regularly checked for mycoplasma contamination, and only passages proven to be free from mycoplasma were used for in vitro and in vivo experiments.

#### 4.2.1. Viral Transduction of CHO-K1 Cells for GRPR or PSMA Overexpression

Plasmids used for overexpression of human FOLH1 and GRPR were obtained from Gentaur GmbH, Germany. Reverse transfection was used for both plasmids for the generation of viral supernatants and subsequent viral transduction of CHO-K1 cells. DNA lipid complexes were generated using Lipofectamine^TM^ 2000 (Invitrogen, Thermo Fisher Scientific Inc., Waltham, MA, USA), diluted, and added to HEK293T cells. Cells were incubated for 72 h, and then the supernatant was concentrated using the PEG virus precipitation kit (abcam, Cambridge, UK) according to the manufacturer’s instructions. Concentrated viral particles were stored at −80 °C until further use. The transduction of CHO-K1 cells was conducted in 6-well plates using 7.5 µL concentrated viral particles and 8 µg/mL polybrene per 1 × 10^6^ CHO-K1 cells. After centrifugation, cells were incubated for 72 h at 37 °C before adding puromycin (10 µg/mL) for selection of GFP-positive clones. After 7 days, cells were sub-cultivated using 5 µg/mL puromycin, and single clones were selected. Transgene expression was confirmed by RT-qPCR and/or Western blot. Clones with RT-qPCR/Western blot-confirmed expression of GRPR (clone 3) or PSMA (clone 5) were used for all further experiments. More detailed information on the generation of viral particles and the procedure for stable viral transduction of CHO-K1 cells is given in the SI.

#### 4.2.2. Internalization Experiments

One hour prior to the in vitro experiments, the culture medium (Section 3.4, SI) was replaced with 1.30 mL Ham’s F-12 Nutrient Mix medium supplemented with 1% heat-inactivated FBS, 100 units/mL penicillin and 100 μg/mL streptomycin, and 2.50 μg/mL puromycin. In order to assess the number of cells, three extra wells were seeded for each experiment for parallel cell counting. For this purpose, a LUNA^TM^ automated cell counter and Trypan Blue solution (0.4%, Gibco^®^, Thermo Fisher Scientific Inc., Waltham, MA, USA) were used. The percentage of applied dose was normalized to 10^5^ cells/well for LNCaP and to 10^6^ cells/well for PC-3, CHO-K1, and transduced CHO-K1. For the sake of simplicity, clone 3 of CHO-K1 cells transduced with human GRPR and clone 5 of CHO-K1 cells transduced with human PSMA are reported as CHO-K1-GRPR and CHO-K1-PSMA, respectively.

Internalization studies with CHO-K1-GRPR and -PSMA cells were conducted according to a previously published procedure [49] and are described in more detail in the SI. Additionally, in order to identify potential mutual interferences between [^111^In]In-RM2 and [^99m^Tc]Tc-PSMA-I&S, internalization experiments were performed at the 2 h time point with PSMA-I&S (1000-fold excess) instead of the usual BBN blocking agent ([^111^In]In-RM2 on CHO-K1-GRPR cells) and with RM2 (1000-fold excess) instead of 2-PMPA ([^99m^Tc]Tc-PSMA-I&S on CHO-K1-PSMA cells). The amount of radioactivity of each fraction was quantified in a γ-counter and calculated as a percentage of the applied dose (*n* = 3 in triplicate, *n* = 2 for CHO-K1 with [^99m^Tc]Tc-PSMA-I&S).

#### 4.2.3. Saturation Assays

*[^111^In]In-RM2 on CHO-K1-GRPR cells and [^99m^Tc]Tc-PSMA-I&S on CHO-K1-PSMA cells*. Cells were processed as previously published [49]. On the day prior to the experiment, cells (10^6^ cells per well) were seeded in a six-well plate and incubated at 37 °C and 5% CO_2_ overnight, allowing them to attach. The next day, the culture medium was replaced with 0.8 mL Ham’s F 12 Nutrient Mix (1% FBS), and the cells were kept in the fridge for 30 min before the addition of the radioligand. Cells were either incubated with [^111^In]In-RM2 (100 μL/well; 19.0 kBq–1.7 MBq) or [^99m^Tc]Tc-PSMA-I&S (100 µL/well; 3.50–530 kBq) with increasing radioligand concentrations (1, 2.5, 5, 10, 25, 50, and 75 nM in NaCl), depending on the cell line. The saturation assay was performed at 4 °C to avoid internalization. Non-specific binding of [^111^In]In-RM2 was determined in the presence of BBN (5 µM/well for radiopeptide concentrations < 10 nM, and 50 µM/well for higher concentrations) or 2-PMPA (10 µM/well for radiopeptide concentrations < 10 nM, and 100 µM/well for higher concentrations), respectively. Total incubation volume per well: 1 mL. Respective aliquots of the radiolabeled peptides (100 μL; 1–75 nmol, 19 kBq–1.70 MBq for [^111^In]In-RM2 and 3.5–530 kBq for [^99m^Tc]Tc-PSMA-I&S) were prepared in triplicate to assess the applied dose per well. The obtained fractions and standard aliquots were measured in a γ-counter. Dissociation constants (K_d_) and maximum receptor occupancy (B_max_) were calculated from the data for specific binding with non-linear regression using GraphPad Prism 8 (*n* = 3–4 in triplicate).

### 4.3. γ-Counter Set-Up for Dual-Isotope Measurements

The energy resolution of the γ-counter (NaI-based detector) did not allow for nuclide-pure quantification. Mainly, crosstalk of the ^111^In 171 keV photopeak into the ^99m^Tc photopeak window disallowed for a direct quantification of ^99m^Tc and ^111^In in two consecutive measurements. Therefore, the probes were measured by an open window protocol (15–2000 keV, no decay correction, no background correction, 60 s per probe) for several times over a period of 2–3 weeks to use these datasets in specific calculation methods (based on the radioactive decay of the radionuclides) to obtain the correct activity for each individual radionuclide at the start of the measurement series. A detailed description of the exact quantification protocol is given in the SI (Section 3.6). Prior to any measurement of dual-tracer biodistribution probes (Section 4.5.2), several probes with predefined activities (5 kBq and 50 kBq) and different activity ratios of ^111^In and ^99m^Tc (^111^In/^99m^Tc: 0%/100%, 5%/95%, 25%/75%, 50%/50%, 75%/25%, 95%/5%, 100%/0%) were measured and evaluated to ensure correct quantification by this γ-counter set-up, measuring and analysis protocol. The respective results are given in the SI (Section 3.5).

As with single-tracer studies, conversion factors must also be determined for dual-tracer studies to allow for correct conversion of the obtained CPM into MBq, which in turn enables calculation of the respective % ID/g values. Conversion factors for both ^111^In and ^99m^Tc were defined by external standards (3 × 1.5 kBq, 3 × 15 kBq and 3 × 150 kBq for each isotope, 1 mL/standard, same vials as used for biodistribution to ensure the same measuring geometry), which were measured some days prior to the biodistribution probes. The resulting CPM/MBq values for ^111^In and ^99m^Tc were considered as constant for the following weeks in which the probes were measured. Measurement of (internal) standards together with the probes, as it can be realized for single-tracer studies, was not conducted, since the preparative and calculation effort was considered too cumbersome in view of the already escalating % ID/g calculation procedure for dual-tracer biodistribution probes. However, it is recommended to repeat external standard measurements after some weeks or months, or in any case shortly before starting the next dual-tracer measurement series, to be able to correct for deviations due to changes in temperature, geometry, or technical updates of the γ-counter device.

### 4.4. Dual-Isotope SPECT (Phantom) Measurements

All measurements were performed on a small animal multimodality scanner (Siemens Inveon^®^ PET/SPECT/CT, Knoxville, TN, USA) using a mouse whole body multi-pinhole collimator (5 × 1 mm). The SPECT modality comprises two gamma detectors, each with a 68 × 68 array of thallium-doped sodium iodide crystals (2.2 mm × 2.2 mm each), resulting in a total scan area of 150 mm × 150 mm and a dynamic range of 30–300 keV. SPECT data could not be acquired in list-mode format (due to technical limitations), but specific energy windows needed to be chosen ahead. The respective calibration factor, either for ^99m^Tc or ^111^In, is directly applied to the acquired dataset. As a consequence, data from one scan cannot be processed into different images by choosing different energy windows after image acquisition. In contrast, the images needed to be acquired sequentially, shortly after each other. This procedure was implemented since a simultaneous measurement was not possible due to these technical constraints but was considered as quasi-simultaneous in case of short SPECT scans (≤20 min). A typical scan sequence started with a 7 min CT, followed by a 10 min SPECT scan using the ^99m^Tc window prior to a 10 min SPECT scan using the ^111^In window (or vice versa).

For single-tracer animals, the respective second SPECT scan was skipped for the nuclide that was not injected, and the SPECT imaging period was increased to up to 60 min (Section 4.5.1).

For both single and dual-tracer SPECT scans, the starting time point of the (first) SPECT scan was defined as the imaging time point.

### 4.5. In/Ex Vivo Evaluation

Six-to-eight-week-old female immunodeficient mice (NOD SCID) were obtained from the Core facility laboratory animal breeding and husbandry of the Medical University of Vienna, Himberg, Austria. Animals were kept in individually ventilated cages with 12 h light–dark cycle. All experiments were approved by the Federal Ministry of Education, Science and Research, Republic of Austria (2020-0.253.820 and BMBWF-66.009/0122-V/3b/2019) and performed according to the guidelines from the Federation of the European Laboratory Animal Science Associations (FELASA). After an acclimatization period of two weeks, animals were injected with 3 × 10^6^ CHO-K1-GRPR cells in the right flank and 3 × 10^6^ CHO-K1-PSMA cells in the left flank (3 × 10^6^ in 100 µL serum-free medium each). It should be noted that approximately the same passage (±1 passage number) was used for inoculation for both cell lines to ensure similar cell viability, target incidence, and density in vivo. Tracer uptake (and thus target expression) was confirmed three days prior to inoculation in cell uptake experiments. Two animals were injected with 5 × 10^6^ PC-3 cells in 100 µL serum-free medium for a control experiment using [^111^In]RM-2. Tumor growth and body weight were monitored daily. Animals were subjected to imaging after 1.5–2 weeks of tumor growth (final tumor volume of 100–800 mm^3^). Endpoints were defined as rapid weight loss up to 20% body weight, tumor volume ≥ 1000 mm^3^, tumor volume < 1000 mm^3^ with signs of necrosis (ulcerated surface), as well as clearly detectable apathy and pain upon touch.

#### 4.5.1. *µ*SPECT/CT Imaging

Xenograft-bearing NOD SCID mice were anesthetized using isoflurane (2.5% in 1.5 mL oxygen) and intravenously injected with 5.22–34.5 MBq (0.37–2.69 nmol) of [^111^In]In-RM2 or 6.00–54.0 MBq (0.16–0.67 nmol) of [^99m^Tc]Tc-PSMA-I&S or both (dual-tracer cocktail) via the tail vein (max. injection volume 150 µL) and returned to their cages until the imaging procedure (randomized, non-blinded study design). In a pilot study, [^99m^Tc]Tc-PSMA-I&S SPECT imaging was performed 1 h, 2 h, 4 h, and 6 h p.i., and [^111^In]In-RM2 SPECT imaging was performed at 4 h, 6 h, and 8 h p.i. Animals were placed into the field of view of the scanner (*μ*SPECT/CT Inveon, Siemens Medical Solution, Knoxville, TN, USA) and vital parameters (respiration, body temperature) were continuously monitored using a dedicated device (bioVet; m2m imaging, Cleveland, OH, USA). A 7-minutes cone beam CT was performed followed by a static SPECT acquisition for 10, 30, or 60 min using a multi-pinhole collimator (5 × 1 mm). (^111^In photopeak: 154–188 keV and ^99m^Tc photopeak: 126–154 keV). After SPECT acquisition, mice returned to their cages to be re-imaged at later time points or were sacrificed by cervical dislocation for further use in biodistribution studies. SPECT images were reconstructed using OSEM (6 subsets, 10 views per detector, 16 iterations) and corrected for attenuation (based on the µ-map generated from the CT), scatter, and decay. Rigid-body image registration was performed using the image analysis software PMOD 3.8 (PMOD Technologies Ltd., Zurich, Switzerland).

#### 4.5.2. Biodistribution Studies

Animals used in imaging studies were sacrificed at different time points *post injectionem* (p.i.) after the last imaging time point by cervical dislocation under isofluorane anesthesia. Selected organs were removed and weighed, and organ activities were measured in a γ-counter. For animals in which a dual-tracer cocktail was administered, the organs were measured several times over a period of 2–3 weeks to use these datasets in specific calculation methods (Section 3.6 in the SI) to obtain the correct activity for each individual radionuclide at the start of the measurement series. Deviations between some imaging data (% ID/cc) and biodistribution data (% ID/g) can be observed due to the low resolution of SPECT and concomitant overestimation of activities in small objects, tissue densities other than 1, and no cross-calibration between γ-counter and SPECT scanner. Moreover, in vivo SPECT images include organ perfusion (radioactivity in the blood) while ex vivo organs are usually washed to remove residual blood.

#### 4.5.3. Protein Extraction and Western Blot Analyses of Cell and Tissue Lysates

Preparation of cell pellets and tissue samples, as well as their analysis via Western blotting, is described in detail in the SI.

## 5. Conclusions

To the best of our knowledge, we hereby present the first combinatory use of [^99m^Tc]Tc-PSMA-I&S and [^111^In]In-RM2 in a dual-tracer approach to improve PCa diagnosis. With the aim of developing a valid and reliable in vitro and in vivo model for the investigation of the dual-tracer imaging concept, two PCa-associated targets, PSMA and GRPR, were chosen for a first proof-of-concept study. CHO-K1 cells transduced to stably express the human GRPR and PSMA protein proved to be valuable alternatives to the commonly used LNCaP and PC-3 cell lines for in vitro experiments. Therefore, CHO-K1-GRPR and CHO-K1-PSMA were assumed to represent an appropriate combination for the simultaneous establishment of GRPR^+^ and PSMA^+^ tumor xenografts in a mouse model, since they showed similar growth rate and expression density of the respective target. However, the positive in vitro results obtained with [^99m^Tc]Tc-PSMA-I&S and [^111^In]In-RM2 could not be transferred to the in vivo situation, most likely due to loss of target expression. Therefore, we need to emphasize that the novel transduced cell lines, developed specifically for this project, are not suitable to generate an in vivo model for PSMA/GRPR-targeting dual-tracer studies. A general word of caution needs to be added that results from an in vitro model may not necessarily be successfully transferred to an in vivo setting. Ideally, for future experiments focusing on dual-tracer approaches, the experimental methods should already be established to ensure the validity of the data generated by novel dual-tracer concepts and to allow for a better comparison to single-tracer data. Based on the results obtained by this study, we recommend the use of alternative PCa-associated in vivo models based on cell lines that are also known to preserve high biomarker expression in their xenografts in vivo (e.g., LNCaP for PSMA expression and PC-3 for GRPR expression instead of transduced cell lines). We propose that such a combination could lead to a more positive outcome of the study with [^99m^Tc]Tc-PSMA-I&S and [^111^In]In-RM2 as a dual-tracer cocktail. However, this comes at the cost of a more elaborate procedure required for dual xenograft generation. Along with the strategies developed during this project to address critical scientific and technical aspects, we aim to pave the way for future, optimized dual-tracer SPECT applications. We want to share our knowledge on potential challenges that need to be considered when aiming to realize such a dual-tracer concept with the scientific community.

## Figures and Tables

**Table 1:** In vitro data of [^111^In]In-RM2 and [^99m^Tc]Tc-PSMA-I&S determined by internalization experiments and saturation assays using CHO-K1-GRPR or CHO-K1-PSMA cells.

Compound	Total Cell Uptake % AD/10^6^ Cells ^[a]^	K_d_ [nM] ^[b]^	B_max_ [nM] ^[b]^	N° of Binding Sites per Cell
[^111^In]In-RM2	40.9 ± 2.4	2.33 ± 0.40	0.84 ± 0.03	5.06 × 10^5^
[^99m^Tc]Tc-PSMA-I&S	31.5 ± 1.9	3.84 ± 0.44	0.64 ± 0.02	3.85 × 10^5^

^[a]^ Total cell-bound (membrane-bound + internalized) fraction of [^111^In]In-RM2 and [^99m^Tc]Tc-PSMA-I&S at 2 h. Results are normalized to 10^6^ cells; values are mean ± SD (*n* = 3 in triplicate). ^[b]^ Determined by saturation binding assays; values are given in mean ± SD as calculated by GraphPad Prism 8 (*n* = 3 in triplicate).

## Data Availability

The original contributions presented in the study are included in the article and Supplementary Materials, further inquiries can be directed to the corresponding author.

## Supplementary Materials

The following supporting information can be downloaded at: https://www.mdpi.com/article/10.3390/ph18071002/s1. Figure S1: Chemical structure of the precursor peptide RM2 (1). Figure S2: HPLC quality control of compound 1. Figure S3: LR-MS data of compound 1. Figure S4: HPLC quality control of compound S1. Figure S5.: LR-MS data of compound S1. Figure S6: Compound S4 HPLC quality control. Figure S7: High-resolution MS data of compound S4. Figure S8: NMR data of compound S4. Figure S9: Compound S5 HPLC quality control. Figure S10: LR-MS data of compound S5. Figure S11: Compound S6 HPLC quality control. Figure S12: LR-MS data of compound S6. Figure S13: Compound 2 HPLC quality control. Figure S14: LR-MS data of compound 2. Figure S15: A) Radio-HPLC of [^111^In]In-RM2 and B) UV/Vis HPLC of ^nat^In RM2. Figure S16: Radio-HPLC of [^99m^Tc]Tc-PSMA-I&S. Figure S17: One time point (2 h) internalization assay with [^111^In]In-RM2 and [^99m^Tc]Tc-PSMA-I&S using CHO-K1 cells as a negative control. Figure S18: One time point (2 h) internalization assay with [^99m^Tc]Tc-PSMA-I&S using LNCaP as a positive control. Figure S19: Energy spectra of ^99m^Tc, ^111^In and a mixture of both nuclides. Figure S20: Straight line obtained by plotting the ln(organ, ^111^In) values (y-axis) against Δt. Figure S21: Straight line obtained by plotting the ln(organ, ^99m^Tc) values (y-axis) against Δt. Scheme S1: Synthesis of PSMA-I&S (2) via SPPS and fragment condensation. Table S1: Accuracy of ^99m^Tc activity as determined by specific calculation methods based on continuous γ-counter measurements of ^111^In/^99m^Tc mixtures with predefined isotope ratios using an open window protocol. Table S2: Accuracy of ^111^In activity as determined by specific calculation methods based on continuous γ-counter measurements of ^111^In/^99m^Tc-mixtures with predefined isotope ratios using an open window protocol. Table S3: Exemplary table for the calculation of A_0_(^111^In) for any organ or body fluid in dual tracer experiments. Table S4: Biodistribution data of [^111^In]In-RM2 at different time points, with different amounts of substance and different activities injected (5.22–34.5 MBq) in female tumor xenograft-bearing NOD SCID mice. Table S5: Biodistribution data of [^99m^Tc]Tc-PSMA-I&S at different time points, with different amounts of substance and different activities injected (6.00–54.0 MBq) in female tumor xenograft-bearing NOD SCID mice. References [50,51] are cited in the Supplementary Materials.

## Abbreviations

2-PMPA: 2-Phosphonomethyl pentanedioic acid; AD: Applied dose; A_m_: Molar activity; BBN: Bombesin; BCA: Bicinchoninic acid; B_max_: Maximal number of binding sites; BSA: Bovine serum albumin; CHO: Chinese hamster ovary; CZT: Cadmium–Zinc–Telluride; DI: Dual-isotope; DNA: Deoxyribonucleic acid; DTT: Dithiothreitol; FBS: Fetal bovine serum; FOLH1: Folate hydrolase 1; GCP II: Glutamate carboxypeptidase II; GFP: Green fluorescent protein; GRPR: Gastrin-releasing peptide receptor; HRP: Horseradish peroxidase; i.v.: intravenous; K_d_: (equilibrium) Dissociation constant; mAb: Monoclonal antibody; NOD: Non-obese diabetic; pAb: Polyclonal antibody; PCa: Prostate cancer; PSMA: Prostate-specific membrane antigen; RCP: Radiochemical purity; RCY: Radiochemical yield; RIPA: Radioimmunoprecipitation assay; RLT: Radioligand therapy; RP-HPLC: Reversed-phase high-performance liquid chromatography; RPMI: Roswell Park Memorial Institute; R_t_: retention time; RT-qPCR: Reverse transcription quantitative polymerase chain reaction; SCID: Severe combined immunodeficiency; SDS-PAGE: Sodium dodecyl sulfate polyacrylamide gel electrophoresis; SI: Supporting Information; SPECT: Single-photon emission computed tomography; SPPS: Solid-phase peptide synthesis; TBS-T: Tris-buffered saline with Tween 20; TPS: Total protein staining; Tris: Tris(hydroxymethyl)aminomethane.
