# Supplementary material for: Towards Dual-Tracer SPECT for Prostate Cancer Imaging Using [99mTc]Tc-PSMA-I&S and [111In]In-RM2
Source: Pharmaceuticals (Basel). 2025 Jul 3;18(7):1002. doi: 10.3390/ph18071002 (PMC12298007; doi:10.3390/ph18071002)
Supplement: Supplementary file 1 [file pharmaceuticals-18-01002-s001.zip › pharmaceuticals-3640643-supplementary.pdf]

Towards Dual-Tracer SPECT for Prostate Cancer Imaging Using [^{99m}Tc]Tc-PSMA-I&S and [^{111}In]In-RM2

- Supporting Information -

Carolina Giammei [1,2,3,4,†], Theresa Balber [1,3,5,†], Veronika Felber [1,2,†], Thomas Dillinger [1,6], Jens Cardinale [1], Marie R. Brandt [1,2,3], Anna Stingeder [1], Markus Mitterhauser [1,3,5], Gerda Egger [1,6] and Thomas L. Mindt [1,2,3,5,*]

[1] Ludwig Boltzmann Institute Applied Diagnostics, General Hospital of Vienna, c/o Sekretariat Nuklearmedizin, Währiger Gürtel 18-20, 1090 Vienna, Austria; carolina.giammei@lbiad.lbg.ac.at (C.G.); theresa.balber@meduniwien.ac.at (T.B.); vroni.felber@tum.de (V.F.); thomas.dillinger@lbiad.lbg.ac.at (T.D.); jens.cardinale@lbiad.lbg.ac.at (J.C.); marie.brandt@meduniwien.ac.at (M.R.B.); anna.stingeder@lbiad.lbg.ac.at (A.S.); markus.mitterhauser@meduniwien.ac.at (M.M.); gerda.egger@meduniwien.ac.at (G.E.)

[2] Institute of Inorganic Chemistry, Faculty of Chemistry, University of Vienna, Währinger Straße 42, 1090 Vienna, Austria

[3] Division of Nuclear Medicine, Department of Biomedical Imaging and Image-Guided Therapy, Medical University of Vienna, Währinger Gürtel 18-20, 1090 Vienna, Austria

[4] Vienna Doctoral School in Chemistry, University of Vienna, Währinger Straße 42, 1090 Vienna, Austria

[5] Joint Applied Medicinal Radiochemistry Facility, University of Vienna, Währinger Straße 42, and Medical University of Vienna, Währinger Gürtel 18-20, 1090 Vienna, Austria

[6] Department of Pathology, Medical University of Vienna, Währinger Gürtel 18-20, 1090 Vienna, Austria

* Correspondence: thomas.mindt@univie.ac.at

† These authors contributed equally to this work.

Keywords: dual tracer approach; indium-111; technetium-99m; SPECT; GRPR; PSMA; tumor heterogeneity; CHO-K1-GRPR; CHO-K1-PSMA

1 GENERAL INFORMATION

Solvents and all other chemicals were purchased from B. Braun, Sigma-Aldrich, Merck, and Carl Roth in the quality grade "for synthesis". Buffers and stock solutions were prepared using Millipore water. Protein LoBind® tubes from Eppendorf were used for syntheses/storage.

Solid phase peptide synthesis (SPPS) was carried out manually using a syringe shaker (Janke&Kunkel Typ VX7). For the synthesis of RM2, Rink Amide MBHA resin was purchased from Merck (Novabiochem®), all protected amino acids and the linker Fmoc-4-APipAc-OH (APipAc) from Iris Biotech. The chelator DOTA-tris(*t*Bu)ester was obtained from CheMatech. For the synthesis of PSMA-I&S, Fmoc-D-Lys(Boc)-Wang resin was purchased from Merck (Novabiochem®), all protected amino acids from Iris Biotech, suberic anhydride from SYNCHEM, Glu-CO-Lys-(tBu)$_3$ester from ABX, and *N*-succinimidyl S-acetylthioacetate (SATA) from Sigma-Aldrich.

[^{99m}Tc]NaTcO$_4$ was eluted from an Ultra-TechneKow or TEKCIS ^{99}Mo/^{99m}Tc generator. [^{111}In]InCl$_3$ was purchased from Curium Netherlands B.V.

The blocking agents used for *in vitro* studies, bombesin (BBN) and 2-(phosphonomethyl)pentanedioic acid (2-PMPA), were obtained from Sigma Aldrich.

2 MATERIALS

Semi-preparative RP-HPLC of the products was performed using a Chromolith® SemiPrep column (RP-18e, 100-10 mm) with a Merck Hitachi L-6200A intelligent pump supplied with Merck Hitachi UV detector L-7400 and Packard Radiomatic Flo-One Beta detector with a BGO cell for radioactivity detection. Alternatively, a VWR Hitachi Chromaster 5160 Pump equipped with VWR Hitachi Chromaster 5310 Column Oven, VWR Hitachi Chromaster 5410 UV Detector and Elysia Raytest Gabi Nova radiodetector was used for semi-preparative RP-HPLC. For data acquisition and gradient control on the VWR system, the Clarity VA Chromatography Software (version 8.1.0.79) was used.

Analytical reversed-phase high performance liquid chromatography (RP-HPLC) was performed by using a Chromolith® Performance column (RP-18e, 100-4.6 mm) on an Agilent system (Vienna, Austria) supplied with Autosampler Agilent 1100 Series, Iso Pump (Isocratic Pump) Agilent 1200 Series G1310A, UV-Monitor Agilent 1200 Series G1314B Variable Wavelength Detector (VWD) and Radioactivity Detector Elysia Raytest Gabi Star. For data

acquisition and gradient control, the GINA Star™ Software (version 5.9) was used. Radiolabeled peptides were analyzed by radio-RP-HPLC using a Chromolith® Performance column (RP-18e, 100-4.6 mm) with a VWR Hitachi Chromaster 5160 Pump equipped with VWR Hitachi Chromaster 5310 Column Oven, VWR Hitachi Chromaster 5410 UV Detector and Elysia Raytest Gabi Nova radiodetector. HPLC solvents were H_2O (A) and ACN (B) each supplemented with 0.1% trifluoroacetic acid (TFA). Individual linear gradients, flow, retention times (R_t), and wavelength (λ) are mentioned in the text.

Low resolution (LR)-MS was performed on a Bruker amaZon speed ETD supplied with ESI ion source and 3D ion trap. High-Resolution Electrospray Ionization (ESI) mass spectra (HR-MS) were recorded on a Bruker maXis UHR-TOF spectrometer.

^{1}H- and ^{13}C-NMR spectra were acquired using either Bruker Avance Neo 500 MHz or Avance III 600 MHz Cryo instruments at 25 °C. ^{1}H- and ^{13}C-NMR spectra were referenced to the residual solvent peak ($CDCl_3$: δ=7.26 ppm and δ=77.16 ppm, respectively). All chemical shifts (δ) are reported in ppm, coupling constants (*J*) are in Hertz (Hz) and are reported to the nearest half integer. COSY, HSQC and HMBC NMR spectra were used to confirm and assign the signals of ^{1}H- and ^{13}C-NMR spectra. Multiplicities are described as follows: singlet (s), doublet (d), triplet (t), multiplet (m)

Activities of the respective probes obtained from saturation assays, internalization assays and biodistribution studies were measured by a 2480 WIZARD^2 automatic γ-counter (PerkinElmer, Waltham, USA) and evaluated with GraphPad PRISM Version 8.

3 METHODS

3.1 Syntheses

3.1.1 Syntheses of precursor compounds

3.1.1.1 RM2 synthesis

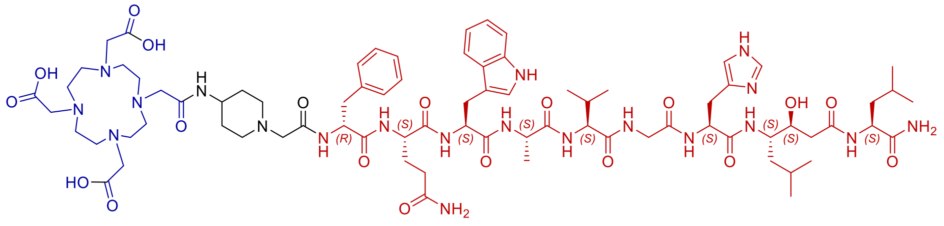

RM2 (1)

Figure S1: Chemical structure of the precursor peptide RM2 (**1**). The GRPR-binding motif is depicted in red and the DOTA chelator is depicted in blue.

RM2 synthesis (**1**, DOTA-APipAc-phe-Gln-Trp-Ala-Val-Gly-His-Sta-Leu-NH_2, *Figure S1*) was accomplished by SPPS on Rink Amide MBHA resin (in accordance with Fmoc/*t*Bu approach of SPPS)[1, 2]. The crude product was purified by RP-HPLC (R_t = 13.24, 80-75% A in 22 min, 4 mL/min, λ = 220 nm). After lyophilization, the product was obtained in a chemical purity of >95% (R_t = 5.94 min, 85% to 60% A in 15 min, 3 mL/min, λ = 220 nm; *Figure S2*), LR-MS: $[M + H^+]^+$ = 1640.99 (calcd for $C_{78}H_{118}N_{20}O_{19}$: 1639.90; *Figure S3*).

3.1.1.2 PSMA-I&S synthesis

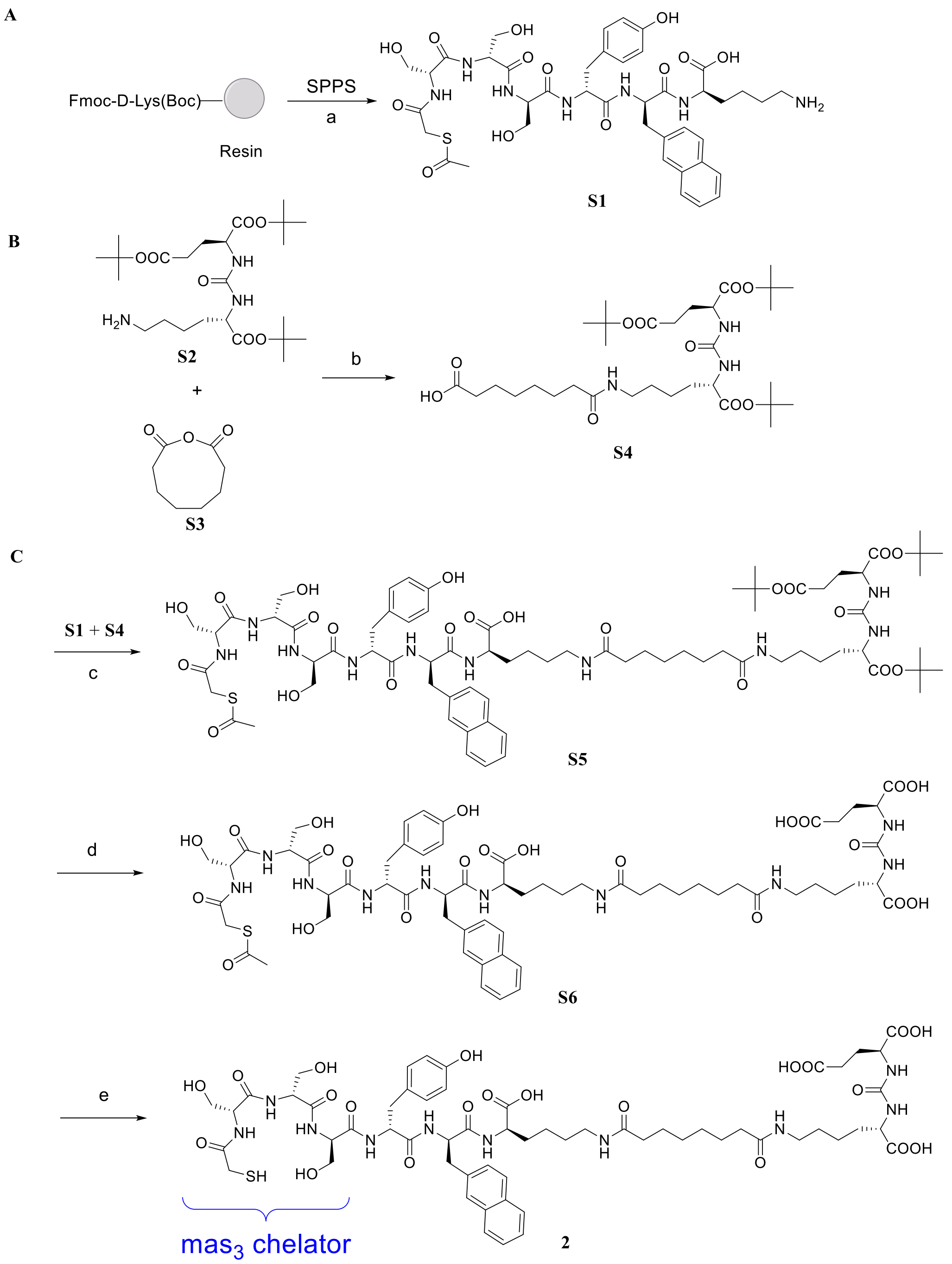

Scheme S1. Synthesis of PSMA-I&S (**2**) *via* SPPS and fragment condensation. A) SPPS; B) b) dry DIPEA, dry ACN, 60 °C, overnight; C) c) dry DMF, HATU, DIPEA, rt, 2 h; d) Cleavage cocktail: TFA/TIPS/H_2O (95:2.5:2.5), 4 h; e) 0.1 M Hydroxylamine, 50 mM EDTA, 0.1 M PB, 0.1 M TCEP, 2-4 h.

Synthesis of PSMA-I&S (**2**, mas3-tyr-nal-lys(Sub-Lys-urea-Glu), *Scheme S1*) was conducted according to published procedures[3,4] with minor modifications. Synthesis of peptide **S1** was accomplished by SPPS using Fmoc-D-Lys(Boc)-Wang resin (in accordance with Fmoc/*t*Bu approach of SPPS)[1,3,4]. The peptide was purified by RP-HPLC (R_t = 15.19 min, 85 - 75% A in

18 min, 4 mL/min, λ = 220 nm). Furthermore, it was characterized by analytical RP-HPLC achieving a chemical purity of >99% (R_t = 6.10 min, 85 - 75% A in 14 min, 3 mL/min, λ = 220 nm; see *Figure S4*) and LR-MS: $[M + H^+]^+$ = 884.36 (calcd for $C_{41}H_{53}N_7O_{13}S$: 884.35; see *Figure S5*). After lyophilization the pure product was obtained.

Synthesis of compound **S4**. Glu-CO-Lys-(*t*Bu)$_3$ester **S2** (1.00 equiv., 50.0 mg, 0.10 mmol) and suberic anhydride **S3** (6.20 equiv., 96.1 mg, 0.62 mmol) were dissolved in dry ACN (250 µL and 3.75 mL, respectively). The two solutions were combined and DIPEA was added (6.20 equiv., 107 µL, 0.62 mmol). The reaction mixture was heated to 60 °C for 20-22 h. It was purified by RP-HPLC (R_t = 12.09 min, 75% - 30% A in 15 min, 4 mL/min, λ = 220 nm). The pure product **S4** was obtained after lyophilization and analyzed via RP-HPLC reaching a chemical purity of ~85% (R_t = 6.20 min, 75 - 20% A in 15 min, 3 mL/min, λ = 220 nm; *Figure S6*). LR-MS: $[M + Na^+]^+$ = 666.3936 (calcd for $C_{32}H_{57}N_3O_{10}$: 644.4120; *Figure S7A*), $[M – H^-]^-$ = 642.3971 (*Figure S7B*). The compound was fully characterized by NMR.

^{1}H-NMR (700 MHz, $CDCl_3$), δ [ppm]: 6.31 (s, 1H), 5.64 (s, 1H), 5.63 (s, 1H), 4.37 – 4.24 (m, 2H), 3.34 (td, *J* = 12.9, 6.3 Hz, 1H), 3.12 (td, *J* = 11.6, 5.6 Hz, 1H), 2.39 – 2.26 (m, 4H), 2.20 (t, *J* = 7.1 Hz, 2H), 2.07 (tt, *J* = 9.0, 6.1 Hz, 1H), 1.83 (m, 2H), 1.72 (dp, *J* = 14.0, 7.0 Hz, 1H), 1.66 – 1.56 (m, 4H), 1.55 – 1.49 (m, 2H), 1.45 (s, 9H), 1.45 (s, 9H), 1.43 (s, 9H), 1.41 – 1.38 (m, 2H), 1.34 (dd, *J* = 11.9, 5.7 Hz, 4H); (*Figure S8A*)

DEPTQ-135 NMR (176 MHz, $CDCl_3$), δ [ppm]: 177.18, 173.86, 172.86, 172.83, 157.79, 82.38, 82.21, 81.03, 53.54, 53.39, 38.94, 36.03, 33.61, 32.35, 31.90, 28.64, 28.28, 28.23, 28.21, 28.15, 28.14, 28.11, 25.43, 24.43, 22.14; (*Figure S8B*).

Synthesis of compound **S5**. Compound **S4** (1.10 equiv., 4.94 mg, 7.70 µmol) and HATU (1.00 equiv., 2.63 mg, 6.93 µmol) were dissolved in dry DMF (300 µL and 30.0 µL, respectively). Compound **S4** was then activated by adding DIPEA (3.30 equiv., 3.89 µL, 22.9 µmol) and the mixture was incubated for 20 min. The activated compound **S4** was added to peptide **S1** (1.10 equiv., 6.78 mg, 7.70 µmol) and the coupling proceeded for 2 h at r.t. The mixture was purified by RP-HPLC (R_t = 12.71 min, 85 - 30% A in 15 min, 4 mL/min, λ = 254 nm; *Figure S9*). The fractions containing compound **S5** were lyophilized and analyzed via RP-HPLC (chemical purity >99%, R_t = 8.18 min, 85 - 20% A in 15 min, 3 mL/min, λ = 220 nm; *Figure S9*) and LR-MS: $[M + 2H^+]^{2+}$ = 755.40 (calcd for $C_{73}H_{108}N_{10}O_{22}S$: 1510.75; *Figure S10*).

Synthesis of compound **S6**. The *t*Bu-protective groups of compound **S5** were removed using a cleavage cocktail of TFA/H_2O/TIPS (95/2.5/2.5, 1.3 mL/mg of compound **S5**) for 4 h at r.t. The compound was purified by RP-HPLC (R_t = 8.03 min, 85 - 40% A in 15 min, 4

mL/min, λ = 220 nm). The pure product was obtained after lyophilization and analyzed via RP-HPLC (chemical purity 98.3%, R_t = 5.02 min, 85 - 40% A in 15 min, 3 mL/min, λ = 220 nm; *Figure S11*) and LR-MS: $[M + H^+]^+$ = 1341.57 (calcd for $C_{61}H_{84}N_{10}O_{22}S$: 1341.56; *Figure S12*).

Synthesis of compound **2**. For removal of the acetyl protective group from the thiol functionality, the following mixture was prepared: Hydroxylamine (0.1 M), EDTA (50.0 mM), phosphate buffer (0.10 M Na_2HPO_4/NaH_2PO_4 (1/2 mix) / H_2O = 1/1) and TCEP (0.10 M) were dissolved in 1 mL H_2O as final volume. Compound **S6** was dissolved in H_2O/ACN (1/1, 120 µL/mg) and the cleavage mixture was added (150 µL/mg of compound **S6**). The reaction proceeded at 37 °C for 2 - 4 h. Purification by RP-HPLC (R_t = 11.04 min, 82 - 70% A in 15 min, 4 mL/min, λ = 220 nm) led to pure product **2** as colorless solid with a chemical purity of >99% as determined by analytical RP-HPLC (R_t = 3.22 min, 80 - 70% A in 10 min, 3 mL/min, λ = 220 nm, *Figure S13*). LR-MS: $[M + H^+]^+$ = 1299.54 (calcd for $C_{59}H_{82}N_{10}O_{21}S$: 1299.55; *Figure S14*).

3.1.2 Synthesis of ^{nat}In-RM2

$InCl_3$ (5.00 equiv., 11.1 mg, 0.05 mmol) was dissolved in HCl (0.05 M in H_2O). This solution was mixed with NaOAc (0.3 M in H_2O) in a ratio 5:6 in order to reach a pH = 4.5. An aqueous solution containing RM2 (1.00 equiv., 0.01 mmol, 1.00 M, 10 µL) was added to the mixture and heated at 95 °C for 20 min. The product was purified and analyzed by RP-HPLC (R_t = 6.59 min, 85 - 60% A in 15 min, 3 mL/min, λ = 220 nm, *Figure S15*) and LR-MS: $[M + 2H^+]^{2+}$ = 876.42 (calcd for $C_{78}H_{115}N_{20}O_{19}In$: 1752.78).

3.1.3 Radiosyntheses

Radiosyntheses are described in the main manuscript. Caution: ^{111}In is a γ-emitter with principal energies at 171 keV and 245 keV and a half-life of approx. 67.3 h (2.805 d). ^{99m}Tc is a γ-emitter (140.5 keV) with a half-life of 6.007 h.[5] All experiments involving ^{99m}Tc and ^{111}In were performed in laboratories approved for the handling of the respective radionuclides and appropriate safety procedures were followed all the time to prevent contaminations.

3.2 PSMA-I&S kit formulation

Kits were prepared according to a published procedure[3] with minor modifications.

Reagents:

1) 10 µL sodium phosphate buffer (0.1 M, pH = 7.5).

   Preparation: 310 mg $NaH_2PO_4 \cdot H_2O$ (2.25 mmol) and 1.09 g Na_2HPO_4 anhydrous (7.68 mmol) were dissolved in 100 mL Tracepur®-H_2O

2) 4 µL sodium tartrate buffer (50 mg/mL in 0.5 M NH_4OAc (aq.), pH = 7)

   Preparation: 25 mg $C_4H_4Na_2O_6 \cdot 2H_2O$ (0.11 mmol) were dissolved in 500 µL 0.5 M NH_4OAc (aq.)

3) 5 µL of a freshly prepared, argon-purged Tin(II) chloride solution (4 mg/mL in ascorbic acid/HCl solution):

   Preparation: Ascorbic acid (3 mg/mL) was dissolved in 10 mM HCl (aq.). The resulting solution (1 - 3 mL) was purged with argon. 2 mg $SnCl_2$ (anhydrous, 4 mg/mL) were dissolved in 500 µL of the ascorbic acid/HCl solution. The latter was dissolved 1:5 in H_2O (e.g. 100 µL + 400 µL H_2O) and 5 µL of this final solution were added to the kit.

For the preparation of <u>one</u> kit, solution 1, 2 and 3 are mixed in a 1.5 mL Protein LoBind tube and the final pH of this mixture was 7-7.5 (measured by using one representative kit, that was only used for pH determination and discarded afterwards). After measuring the pH, 5 µL of PSMA-I&S (5 nmol, 1 mM in H_2O) were added to the 1-3 mixture. The kits were frozen at -80 °C, lyophilized, filled with argon and stored at -20 °C. The amounts of the basic solutions mentioned above should be enough to prepare at least 16 kits.

For *in vivo* experiments, modified kits were prepared, containing 2 µL of PSMA-I&S (2 nmol, 1 mM in H_2O) instead of 5 µL. All other solutions were prepared and mixed in analogy to 5 nmol-kits.

3.3 HPLC chromatograms, mass spectra and NMR spectra

Compound **1** (RM2)

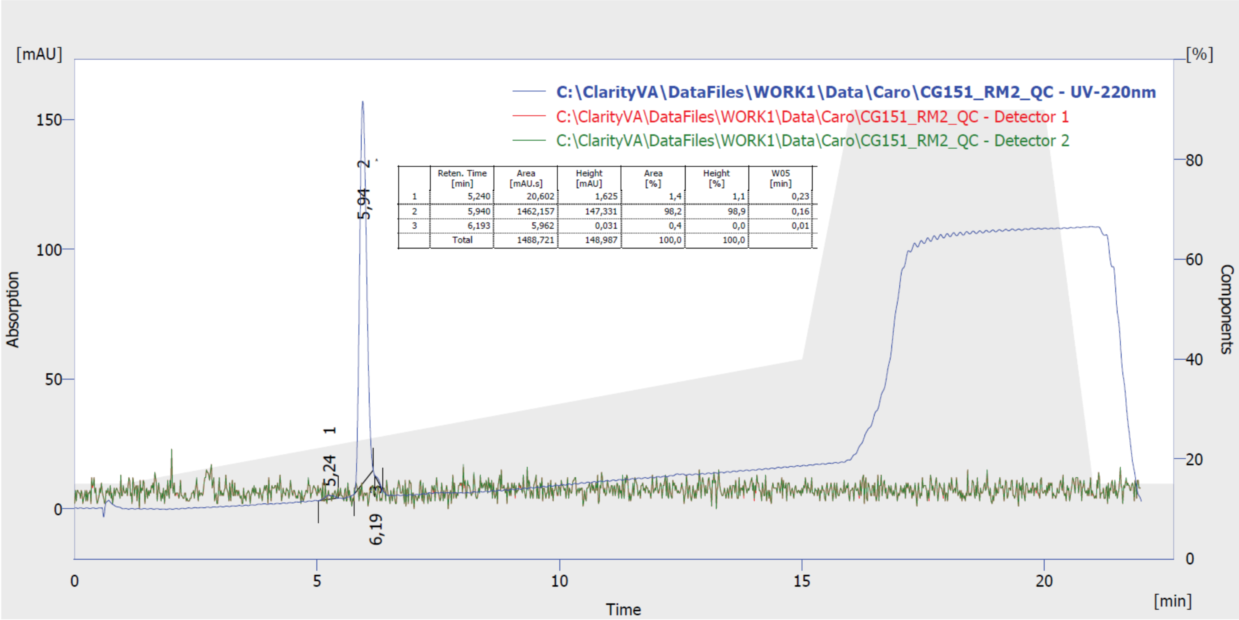

	Reten. Time [min]	Area [mAU.s]	Height [mAU]	Area [%]	Height [%]	W05 [min]
1	5,240	20,602	1,625	1,4	1,1	0,23
2	5,940	1462,157	147,331	98,2	98,9	0,16
3	6,193	5,962	0,031	0,4	0,0	0,01
	Total	1488,721	148,987	100,0	100,0	

Figure S2. HPLC quality control of compound **1**. R_t = 5.94 min, linear gradient from 85% to 60% of eluent A in 15 min with a flow rate of 3 mL/min, λ = 220 nm; Chromolith® Performance (RP-18e, 100-4.6 mm).

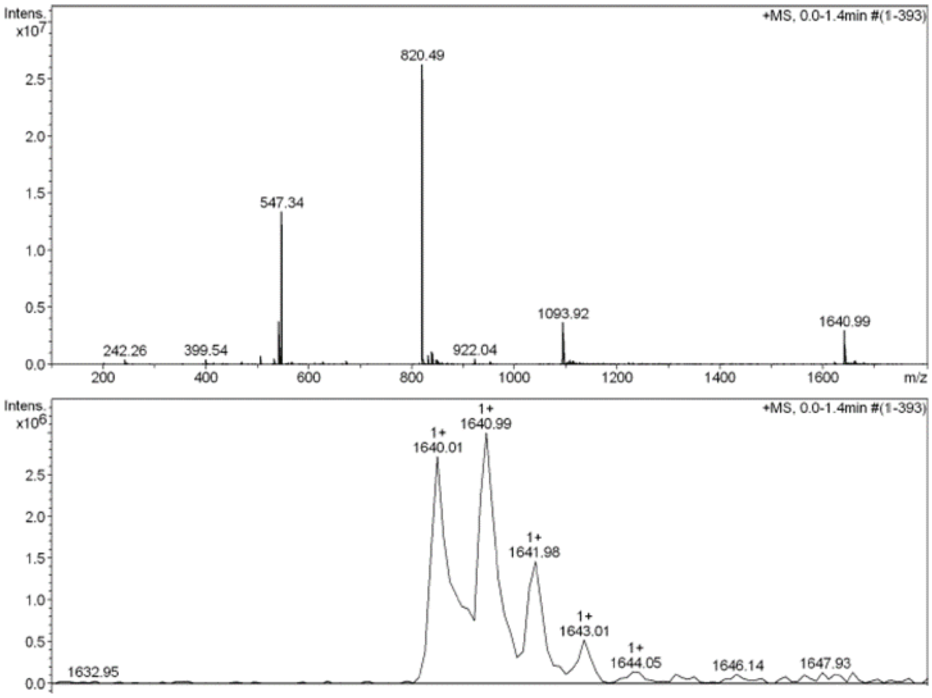

Figure S3. LR-MS data of compound **1**. $[M + H^+]^+$ = 1640.99 (calcd for $C_{78}H_{118}N_{20}O_{19}$: 1639.90).

Compound **S1**

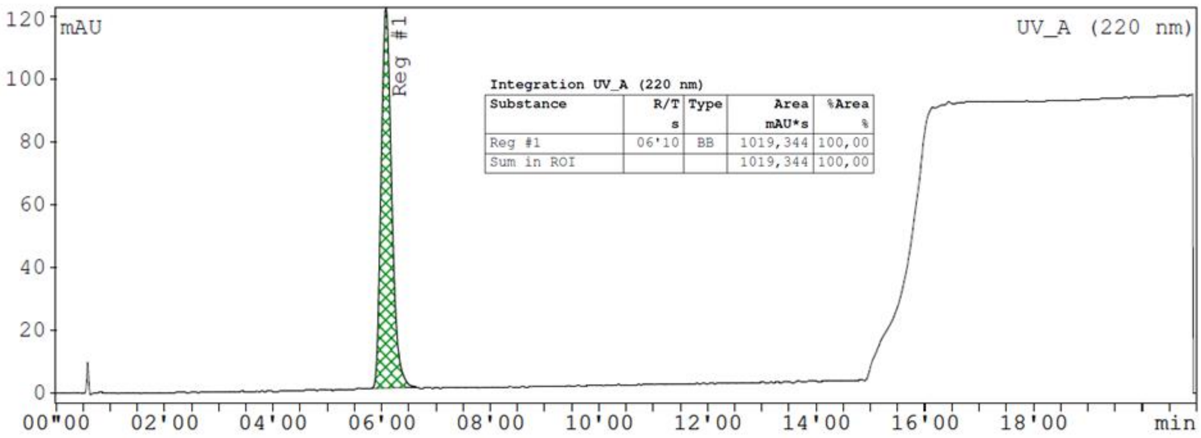

Figure S4. HPLC quality control of compound **S1**. $R_t = 6.10$ min, linear gradient from 85% to 75% of eluent A in 14 min with a flow rate of 3 mL/min, $\lambda = 220$ nm; Chromolith® Performance (RP-18e, 100-4.6 mm).

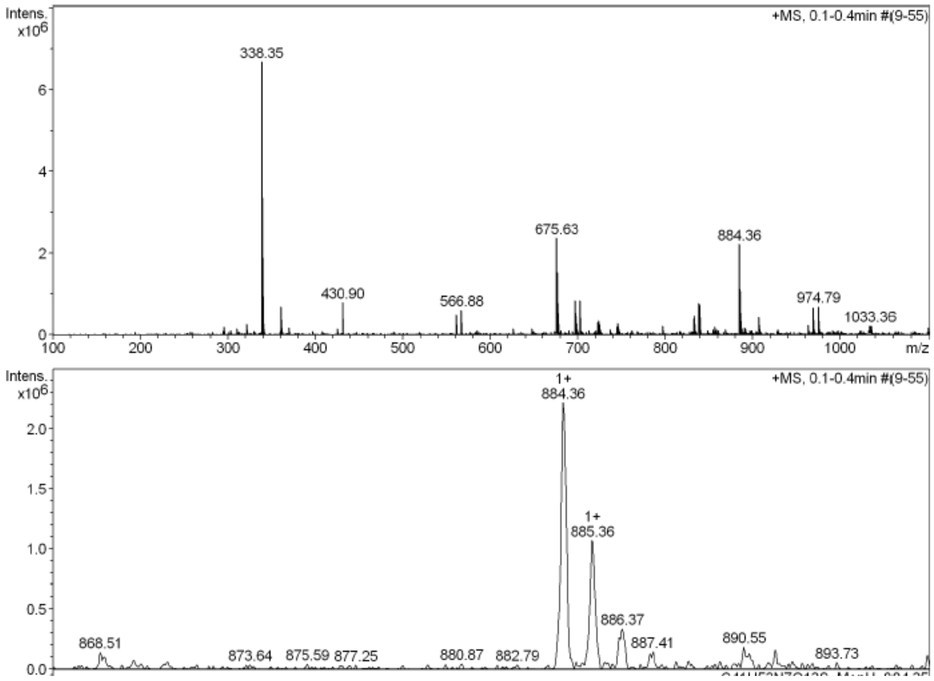

Figure S5. LR-MS data of compound **S1**. $[M + H^+]^+ = 884.36$ (calcd for $C_{41}H_{53}N_7O_{13}S$: 884.35).

Compound **S4**

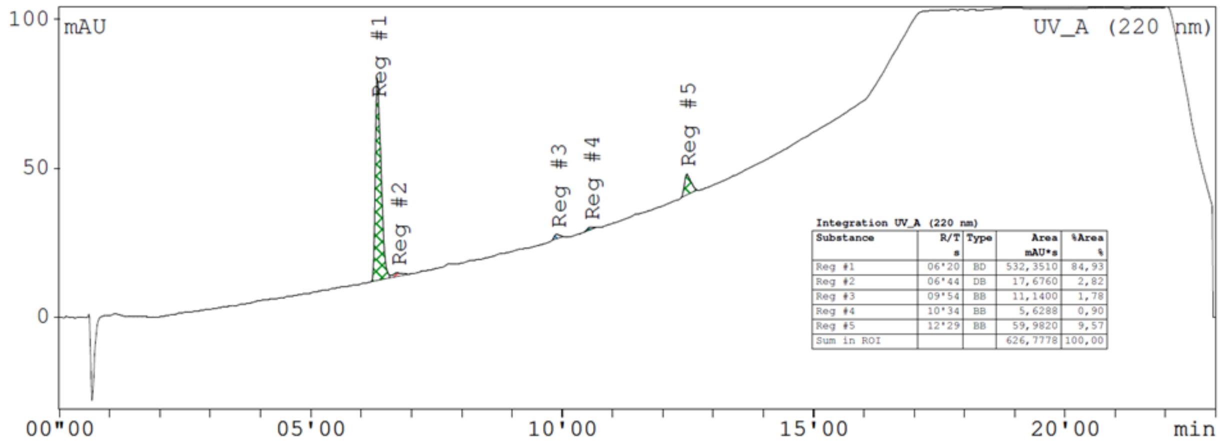

Figure S6. Compound **S4** HPLC quality control. R_t = 6.20 min, linear gradient from 75% to 20% of eluent A in 15 min with a flow rate of 3 mL/min, λ = 220 nm; Chromolith® Performance (RP-18e, 100-4.6 mm).

A)

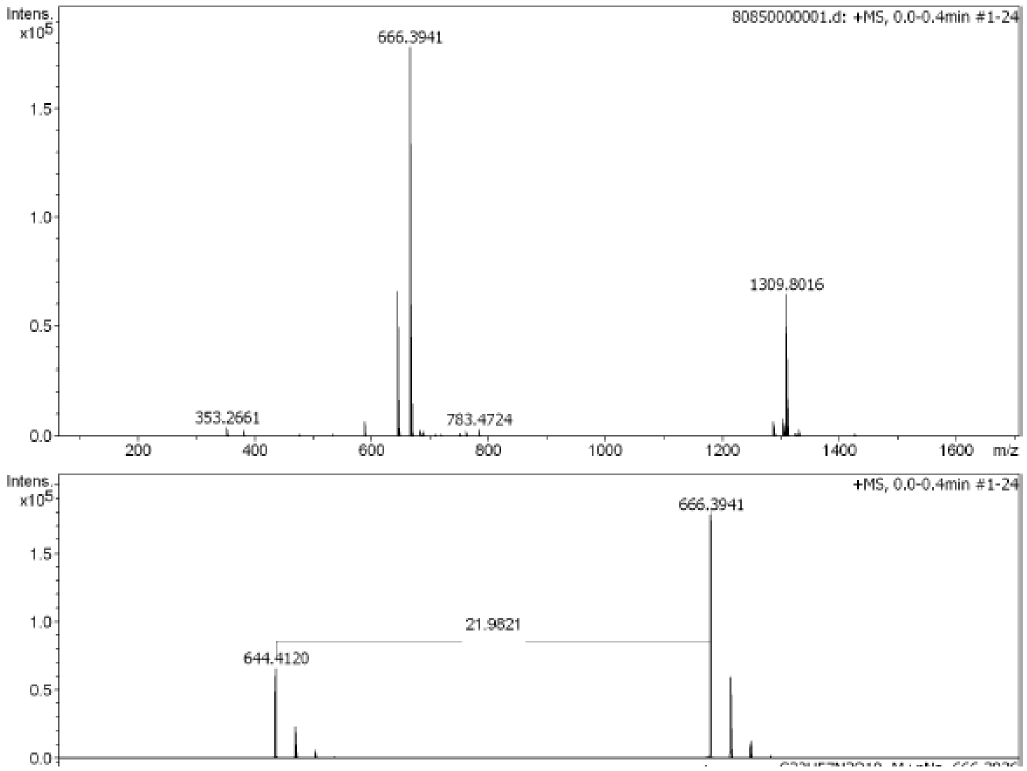

B)

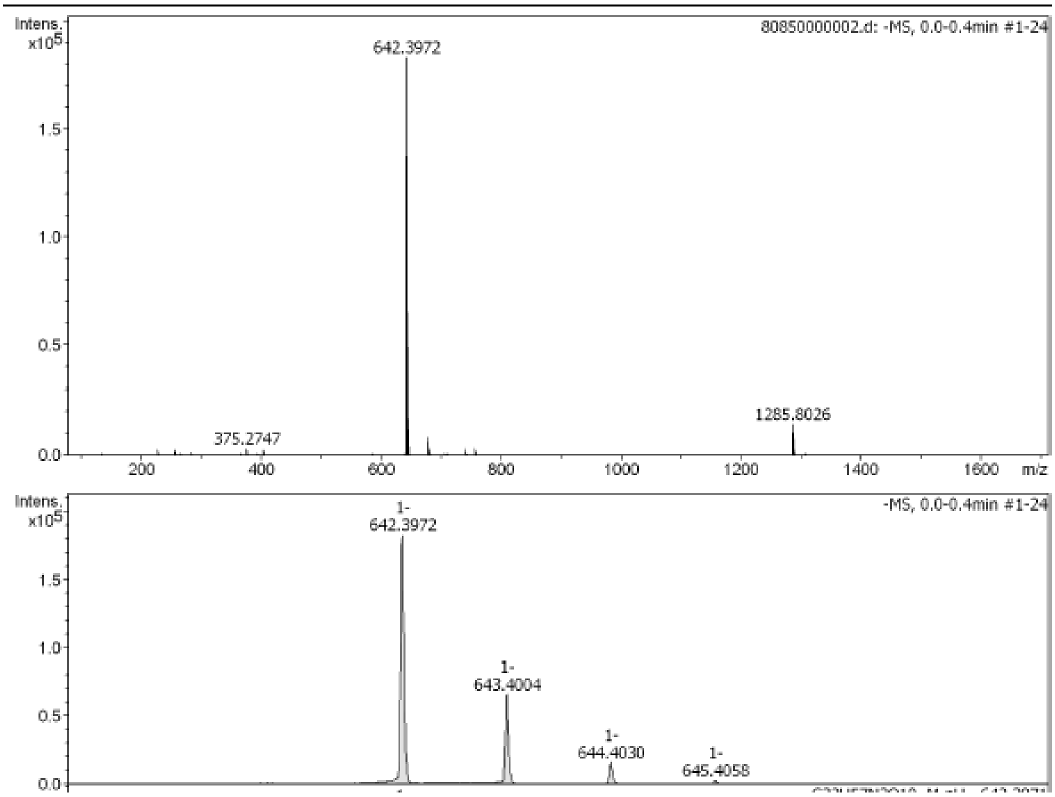

Figure S7. High-resolution MS data of compound **S4**. A) $[M + Na^+]^+$ = 666.3941 (calcd for $C_{32}H_{57}NaN_3O_{10}$: 666.3936); B) $[M – H^-]^-$ = 642.3972 (calcd for $C_{32}H_{56}N_3O_{10}$: 642.3972).

A)

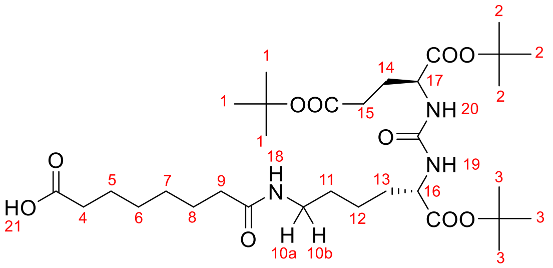

S4

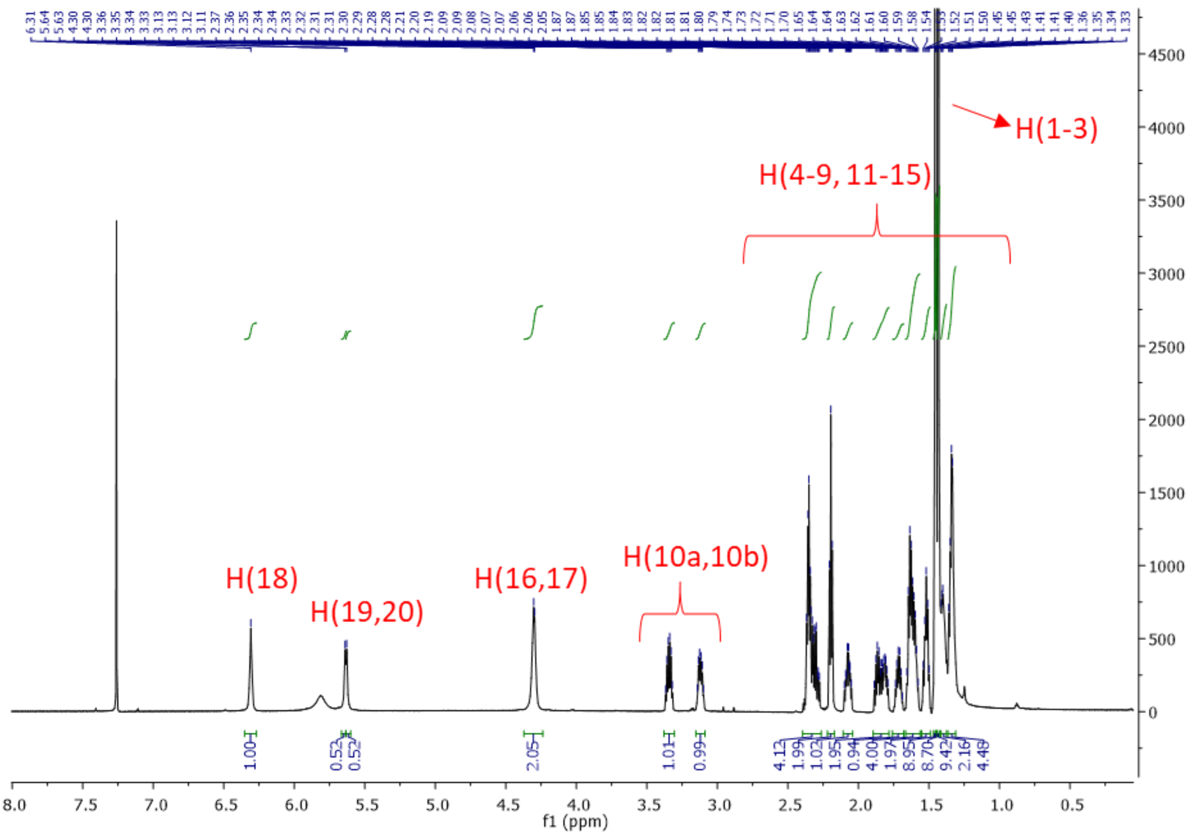

B)

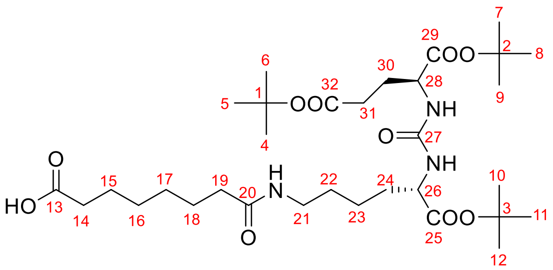

S4

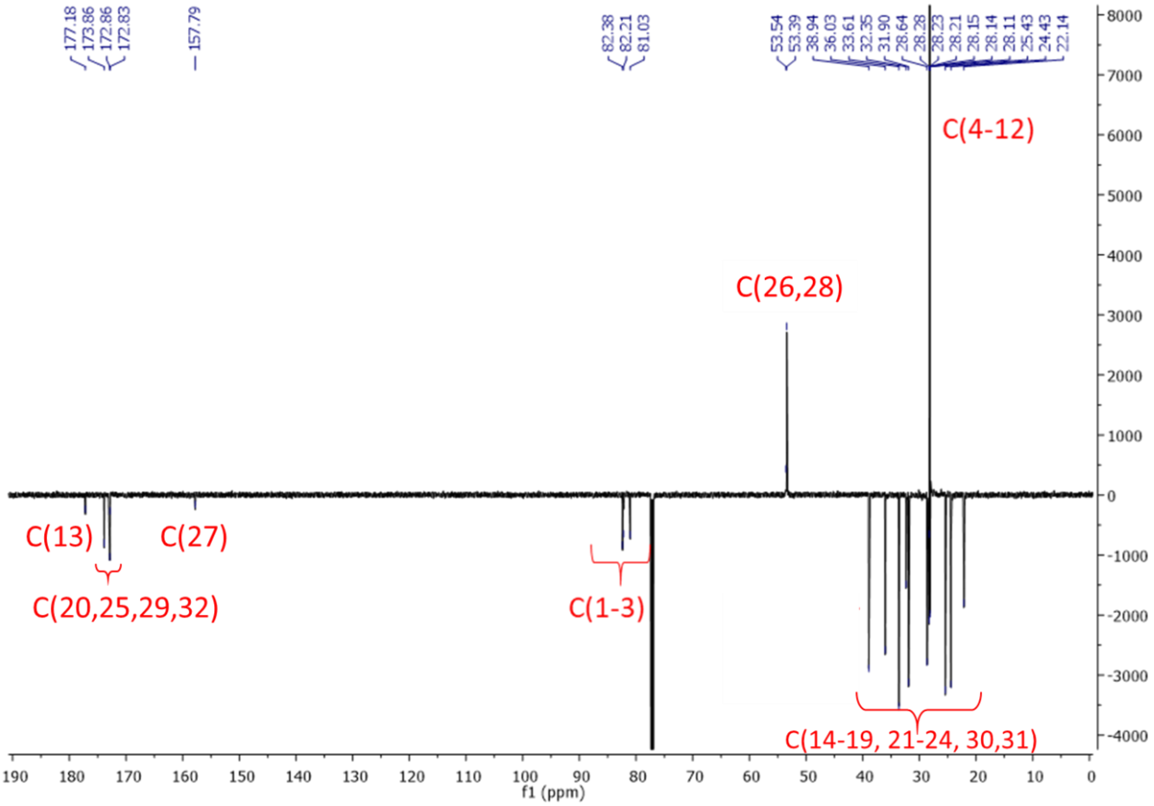

Figure S8. NMR data of compound **S4**. (A) **^{1}H-NMR** (700 MHz, $CDCl_3$) δ [ppm]: 6.31 (s, 1H), 5.64 (s, 1H), 5.63 (s, 1H), 4.37 – 4.24 (m, 2H), 3.34 (td, *J* = 12.9, 6.3 Hz, 1H), 3.12 (td, *J* = 11.6, 5.6 Hz, 1H), 2.39 – 2.26 (m, 4H), 2.20 (t, *J* = 7.1 Hz, 2H), 2.07 (tt, *J* = 9.0, 6.1 Hz, 1H), 1.83 (m, 2H), 1.72 (dp, *J* = 14.0, 7.0 Hz, 1H), 1.66 – 1.56 (m, 4H), 1.55 – 1.49 (m, 2H), 1.45 (s, 9H), 1.45 (s,9H), 1.43 (s, 9H), 1.41 – 1.38 (m, 2H), 1.34 (dd, *J* = 11.9, 5.7 Hz, 4H); (B) **DEPTQ-135 NMR** (176 MHz, $CDCl_3$) δ [ppm]: 177.18, 173.86, 172.86, 172.83, 157.79, 82.38, 82.21, 81.03, 53.54, 53.39, 38.94, 36.03, 33.61, 32.35, 31.90, 28.64, 28.28, 28.23, 28.21, 28.15, 28.14, 28.11, 25.43, 24.43, 22.14.

Compound **S5**

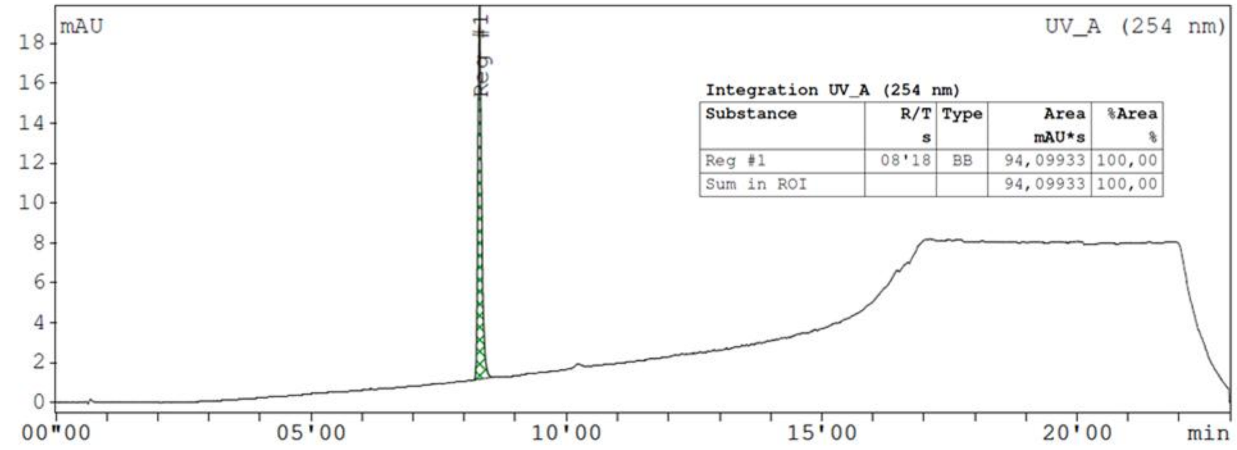

Figure S9. Compound **S5** HPLC quality control. R_t = 8.18 min, linear gradient from 85% to 20% of eluent A in 15 min with a flow rate of 3 mL/min, λ = 254 nm; Chromolith® Performance (RP-18e, 100-4.6 mm).

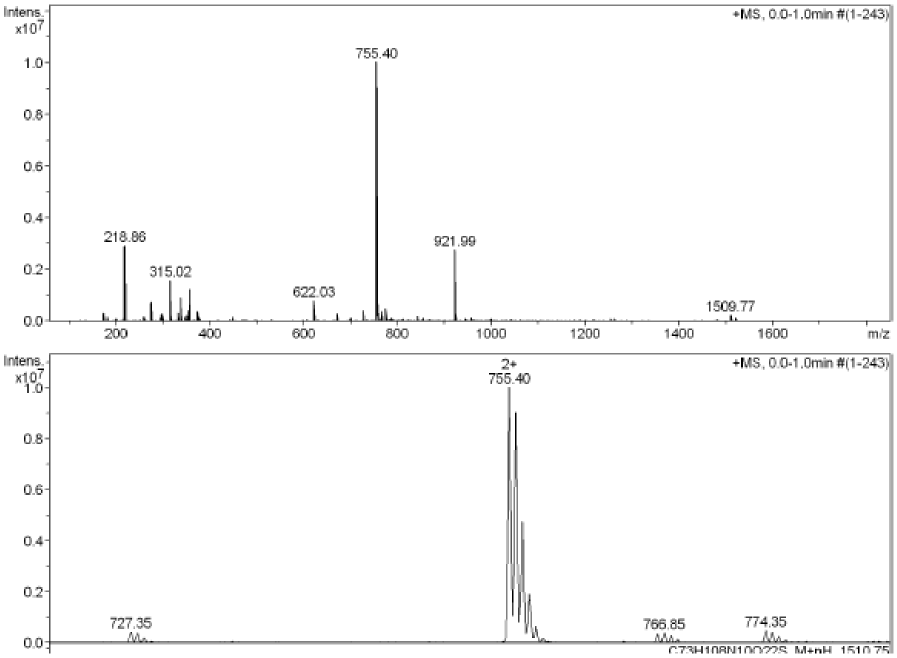

Figure S10. LR-MS data of compound **S5**. $[M + 2H^{+}]^{2+}$ = 755.40 (calcd for $C_{73}H_{108}N_{10}O_{22}S$: 1510.75).

Compound **S6**

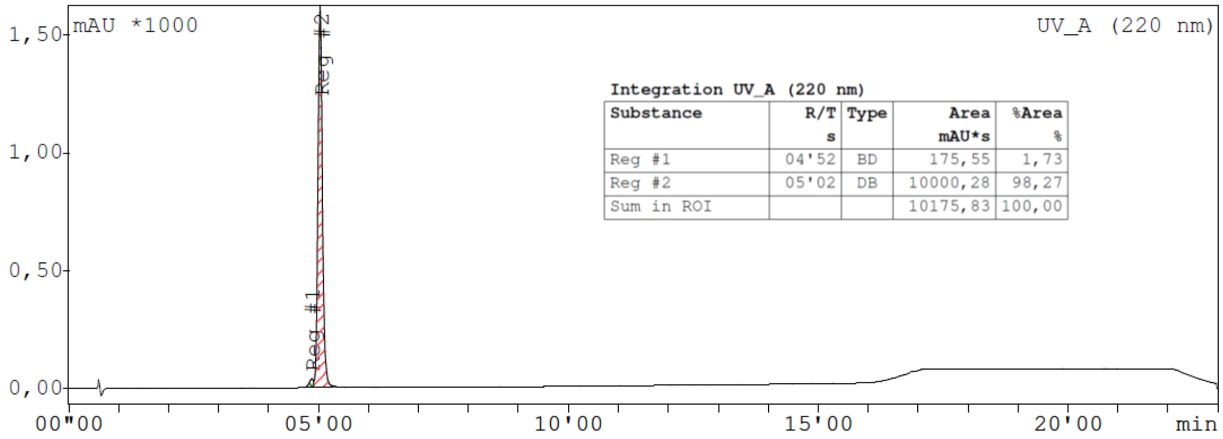

Figure S11. Compound **S6** HPLC quality control. $R_t = 5.02$ min, linear gradient from 85% to 40% of eluent A in 15 min with a flow rate of 3 mL/min, $\lambda = 220$ nm; Chromolith® Performance (RP-18e, 100-4.6 mm).

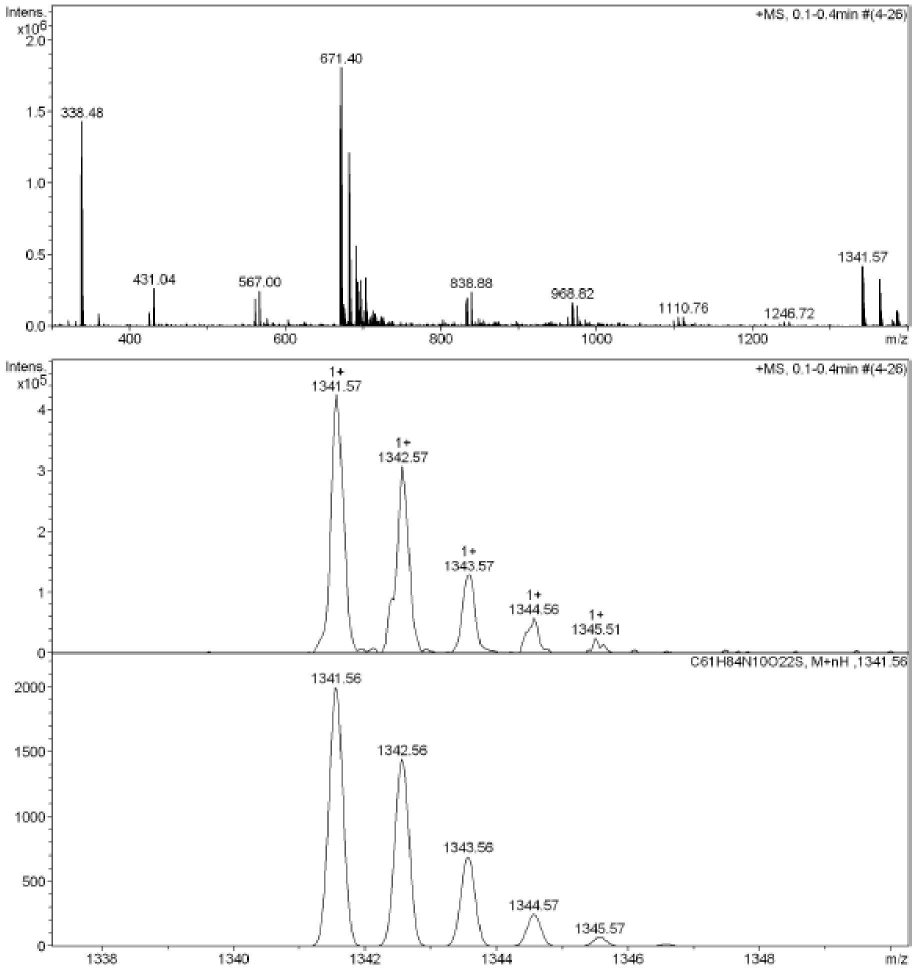

Figure S12. LR-MS data of compound **S6**. $[M + H^+]^+ = 1341.57$ (calcd for $C_{61}H_{84}N_{10}O_{22}S$: 1341.56).

Compound **2** (PSMA-I&S)

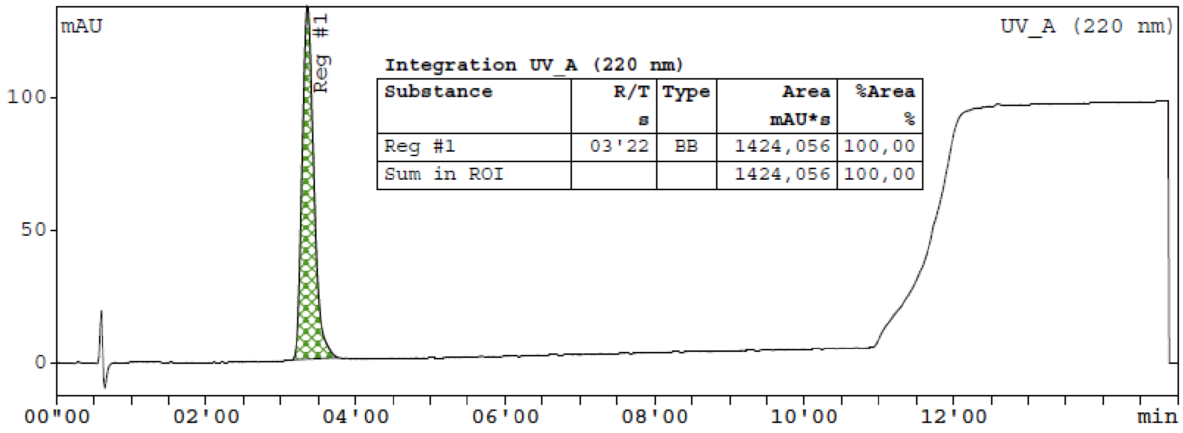

Figure S13. Compound **2** HPLC quality control. R_t = 3.22 min, linear gradient from 80% to 70% of eluent A in 10 min with a flow rate of 3 mL/min, λ = 220 nm; Chromolith® Performance (RP-18e, 100-4.6 mm).

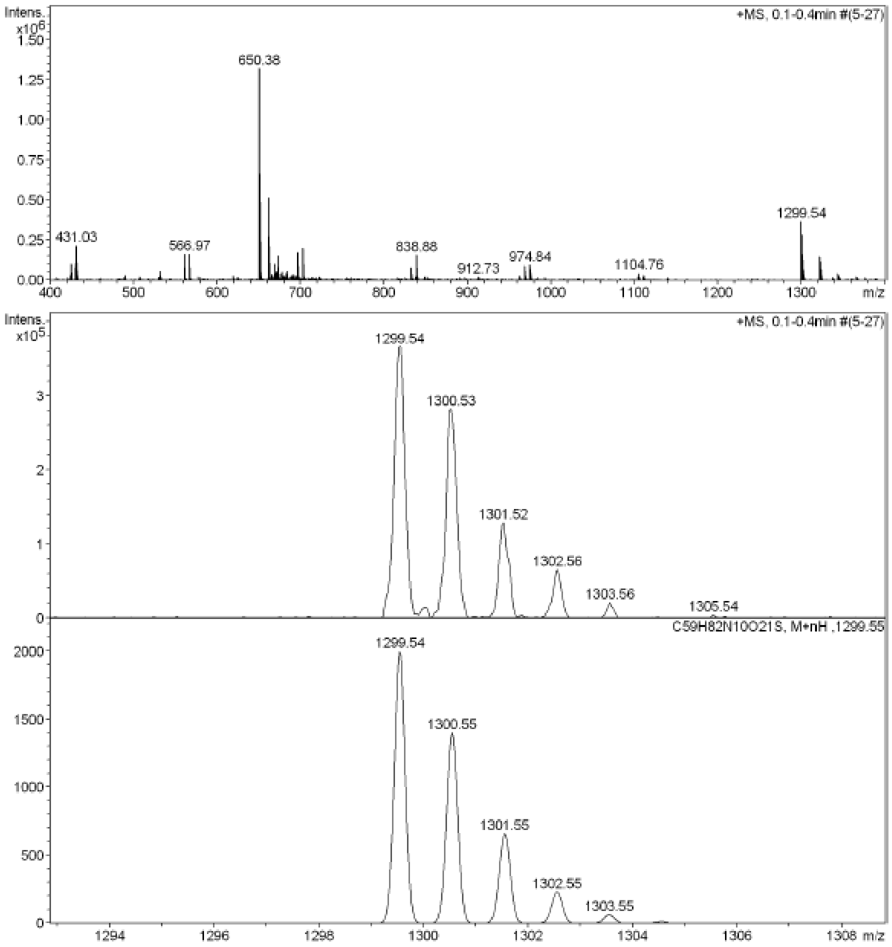

Figure S14. LR-MS data of compound **2**. $[M + H^+]^+$ = 1299.54 (calcd for $C_{59}H_{82}N_{10}O_{21}S$: 1299.55).

Preparation of [^{111}In]In-RM2 ([^{111}In]In-**1**)

A)

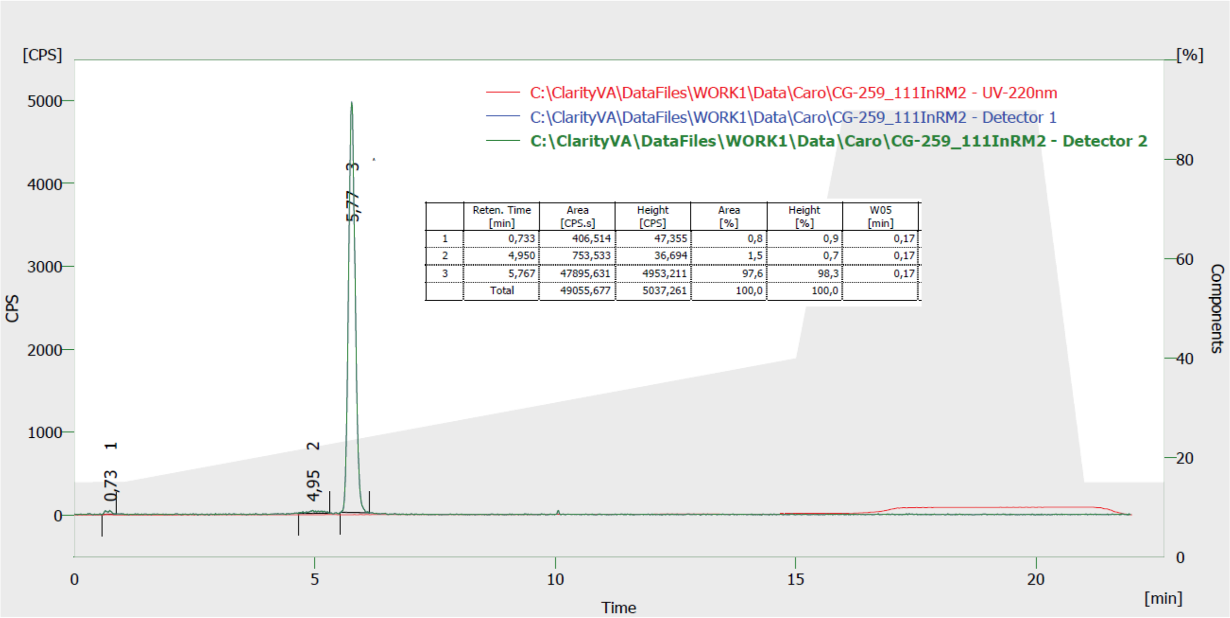

	Reten. Time [min]	Area [CPS.s]	Height [CPS]	Area [%]	Height [%]	W05 [min]
1	0,733	406,514	47,355	0,8	0,9	0,17
2	4,950	753,533	36,694	1,5	0,7	0,17
3	5,767	47895,631	4953,211	97,6	98,3	0,17
	Total	49055,677	5037,261	100,0	100,0	

B)

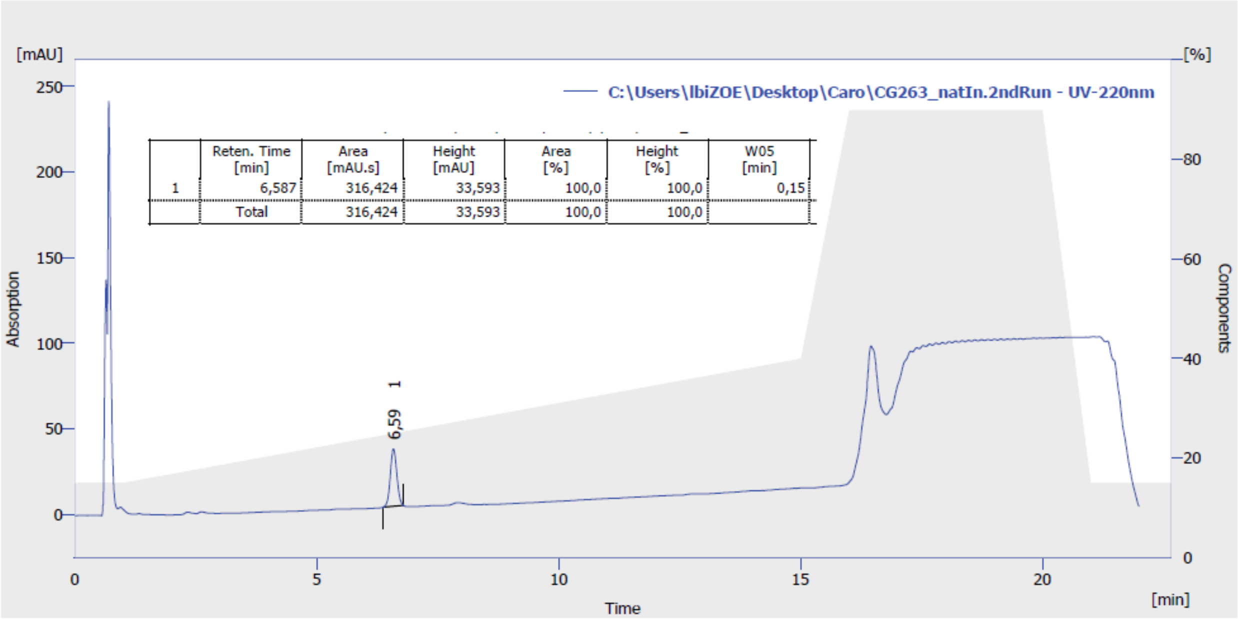

	Reten. Time [min]	Area [mAU.s]	Height [mAU]	Area [%]	Height [%]	W05 [min]
1	6,587	316,424	33,593	100,0	100,0	0,15
	Total	316,424	33,593	100,0	100,0	

Figure S15. A) Radio-HPLC of [^{111}In]In-RM2 (R_t = 5.8 min) and B) UV/Vis HPLC of ^{nat}In-RM2 (R_t = 6.59 min); For both analyses a linear gradient of 85% - 60% A in 15 min was applied with a flow rate of 3 mL/min, λ = 220 nm). Chromolith® Performance (RP-18e, 100-4.6 mm). In this case, the UV/Vis detector was placed downstream after the radiodetector.

Preparation of [^{99m}Tc]Tc-PSMA-I&S ([^{99m}Tc]Tc-**2**)

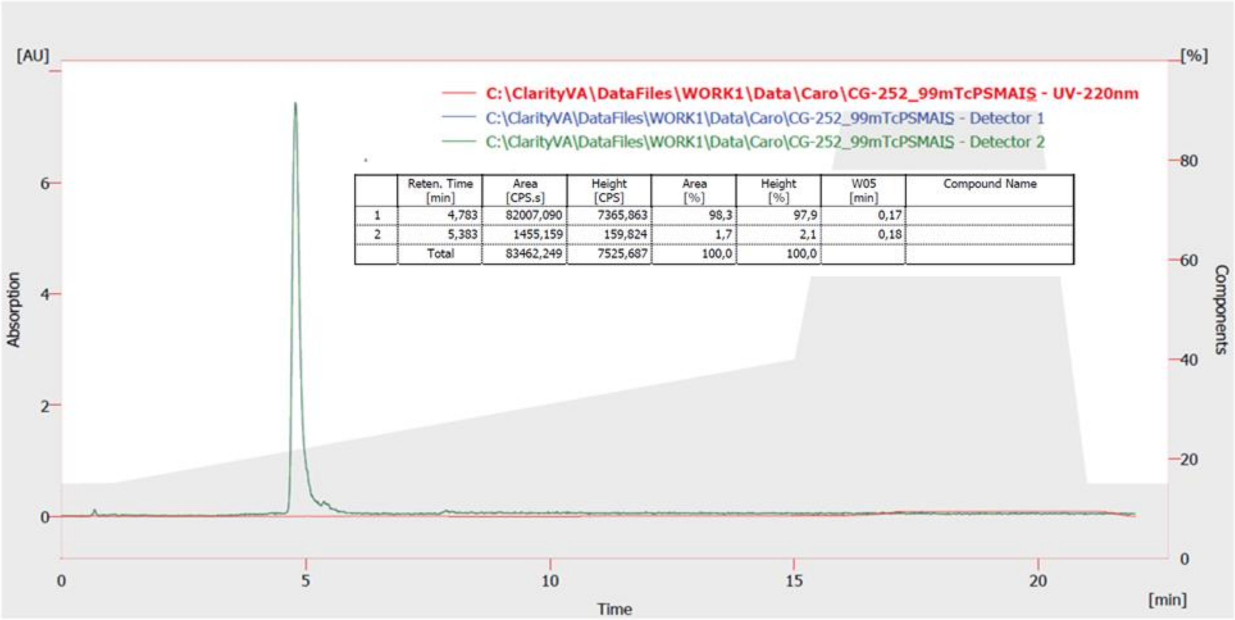

	Reten. Time [min]	Area [CPS.s]	Height [CPS]	Area [%]	Height [%]	W05 [min]	Compound Name
1	4,783	82007,090	7365,863	98,3	97,9	0,17	
2	5,383	1455,159	159,824	1,7	2,1	0,18	
	Total	83462,249	7525,687	100,0	100,0		

Figure S16. Radio-HPLC of [^{99m}Tc]Tc-PSMA-I&S (R_t = 4.78 min, linear gradient of 85% - 60% A in 15 min with a flow rate of 3 mL/min, λ = 220 nm). Chromolith® Performance (RP-18e, 100-4.6 mm).

3.3 Viral transduction of CHO-K1 cells for GRPR or PSMA overexpression

3.3.1 Generation of viral particles

Plasmids used for overexpression of human FOLH1 and GRPR were obtained from Gentaur (Cat.Nr. LV797927 and LV175966). Reverse transfection was used for both plasmids for generation of viral supernatants and subsequent viral transduction of CHO-K1 cells. Lipofectamine™ 2000 (ThermoFisher, Cat.Nr. 11668027) was used to generate DNA-lipid complexes by diluting the target plasmids with the packaging plasmids in a ratio of 2 (FOLH1/GRPR plasmid): 1.25 (psPAX2) : 0.75 (pMD2.G) resulting in a total of 12 µg plasmid DNA in 500 µl of Opti-MEM® I medium without serum. Separately, 50 µl of Lipofectamine™ 2000 was diluted in 500 µl of Opti-MEM® I medium (ThermoFisher, Cat.Nr. 31985070) without serum, mixed gently and incubated at room temperature for 5 minutes. The diluted plasmid DNA was then combined with the diluted Lipofectamine™ 2000 and gently mixed before incubation at room temperature for 20 minutes. While incubation, HEK293FT cells were trypsinized and $3x10^6$ cells were seeded in a 10 cm petri dish containing 10 mL of Opti-MEM® I medium containing 10% FBS but no antibiotics. The DNA-lipid complex was then added to the HEK293T cells and the plate was incubated at 37 °C in a humidified 5% CO_2 incubator. After 24 h, the medium was removed and replaced with 10 mL of DMEM medium

(ThermoFisher, Cat.Nr. 11965092) containing 10% FBS but no antibiotics. Cells were incubated for 48 h before supernatant was harvested and concentrated using the PEG virus precipitation kit (BioVision, Cat.Nr. K904-50) following the instructions of the user manual. Concentrated viral particles were stored at -80 °C until further use.

(psPAX2 was a gift from Didier Trono (Addgene plasmid # 12260 ; http://n2t.net/addgene:12260 ; RRID:Addgene_12260)

(pMD2.G was a gift from Didier Trono (Addgene plasmid # 12259 ; http://n2t.net/addgene:12259 ; RRID:Addgene_12259)

3.3.2 Transduction of CHO-K1 cells

The transduction of CHO-K1 cells with lentiviral vectors was conducted as follows: 1 x 10^6 CHO-K1 cells were seeded into three wells of a 6-well-plate (Corning, Cat.Nr. 3516) in 2.5 mL of Ham's F-12 Nutrient Mix supplemented with 10% FBS. 7.5 µL of concentrated viral particles and 8 µg/mL Polybrene (transfection reagent) were added to each well and mixed by gently swirling the plate. Next, the plates were spun down for 30 minutes at 800 x g at room temperature, before incubating for 72 h at 37 °C. Then 10 µg/mL puromycin was added for selection of GFP positive clones for the next 7 days. Puromycin concentration was then reduced to 5 µg/mL for further subcultivation. For single clone selection, transduced cells were seeded in a low density of 5 x 10^2 cells on a 10 cm dish and grown for 7 days before single clones were picked by trypsinization for further subcultivation and analysis of transgene expression.

3.3.3 Quantitative reverse transcription PCR (RT-qPCR)

RNA from CHO-K1-GRPR and human PC-3 prostate cancer cells was isolated using the RNeasy Mini Kit (Qiagen, Cat.Nr. 74104) following the supplier's protocol including on-column DNaseI digest. 750 ng of isolated RNA was used for reverse transcription by qScript cDNA synthesis kit (Quantabio, Cat.Nr. 95047) following the manufacturer's recommendation. RT-qPCR was conducted with KAPA SYBR® FAST qPCR kits (KAPA Biosystems, Cat.Nr. 07959389001) using 15 ng cDNA per reaction. RT-qPCR was performed on a C1000 thermal cycler, CFX96 real-time system (Biorad) using β-ACTIN as reference gene for normalization. Used primers:

Hamster β-ACTIN forward: AAGGCCAACCGTGAAAAGATG

Hamster β-ACTIN reverse: GCCCTCATAGATGGGCACAG

Human GRPR forward: GCTGCAAACTGATCCCCTTT

Human GRPR reverse: GCAGCATGGAGATGATCCAG

Human β-ACTIN forward: GTCTTCCCCTCCATCG

Human β-ACTIN reverse: AGGGTGAGGATGCCTCTC

3.3.4 Western blot

Total protein was isolated from cell lines as previously described[6]. Pierce™ Coomassie Plus (Bradford) Protein Assay Kit (Thermo Scientific, Cat.Nr. 23236) was used for measuring protein concentrations and 30 µg (for PSMA) or 40 µg (for GRPR) protein per sample was used for SDS-PAGE and Western blot as previously described[6]. PSMA (CST, D4S1F) Rabbit, GRPR (Alomone labs, Cat.Nr. ABR-002) Rabbit, α-Tubulin (Proteintech, Cat.Nr. 66031-1-Ig) Mouse and β-Actin (Proteintech, Cat.Nr. 66009-1-Ig) Mouse antibodies were utilized for detection of protein expression. Secondary antibodies used, included goat anti-rabbit IgG HRP conjugate (JD111036047) and rabbit anti-mouse IgG HRP conjugate (JD315035008).

3.4 *In vitro* evaluation

3.4.1 Calculation of binding sites per cell

For [^{111}In]In-RM2 on CHO-K1-GRPR cells the B_{max} was found to be 0.84 ± 0.03 nM (Figure 6A, Table 1, main manuscript). This B_{max} value corresponds to 5.06 x 10^5 binding sites per cell according to equations 1 - 3:

$$\text{Amount of substance per well: } B_{max} \cdot \text{total incubation volume per well}$$

$$= 0.84 \text{ nM} \cdot 1 \text{ mL} = 0.84 \cdot 10^{-12} \text{ mol} \qquad (1)$$

$$\text{Amount of substance per cell: } \frac{\text{Amount of substance per well}}{\text{Number of cells per well for which } B_{max} \text{ was determined}}$$

$$= \frac{0.84 \cdot 10^{-12} \text{ mol}}{10^6} = 0.84 \cdot 10^{-18} \text{ mol} \qquad (2)$$

With N_A (Avogadro constant) = 6.02214 · 10^{23} mol^{-1}:

$$\textbf{Binding sites per cell}: 0.84 \cdot 10^{-18} \text{ mol} \cdot N_A = \mathbf{5.06 \cdot 10^5} \qquad (3)$$

For [^{99m}Tc]Tc-PSMA-I&S on CHO-K1-PSMA cells the B_{max} was found to be 0.64 ± 0.02 nM (Figure 6B, Table 1, main manuscript). This B_{max} value corresponds to 3.85 x 10^5 binding sites per cell as calculated according to equations 1-3.

3.4.2 Internalization studies

Evaluation of CHO-K1-GRPR with [^{111}In]In-RM2 and CHO-K1-PSMA with [^{99m}Tc]Tc-PSMA-I&S. Approx. 5 x 10^5 CHO-K1-GRPR or CHO-K1-PSMA cells were seeded in Ham's F-12 Nutrient Mix (containing 10% FBS, 100 units/mL penicillin and 100 µg/mL streptomycin, and 2.50 µg/mL puromycin) in 6-well plates and incubated in humidified atmosphere (37 °C, 5% CO_2, 95% air) for 24 h prior to the experiment. Medium was changed to 1.30 mL of Ham's F-12 Nutrient Mix supplemented with 1% FBS one hour before the addition of 100 µL radiolabeled peptide ([^{111}In]In-RM2: 0.17 nmol; 1.50 - 2.00 kBq or [^{99m}Tc]Tc-PSMA-I&S: 0.20 nmol; 8.0 - 14.0 kBq). 1000-fold excess of BBN or 2-PMPA (0.20 µmol; 100 µL per well) were added to determine non-specific binding (blocking experiments). Cells were incubated for 30, 60, 90, 120, and 180 min at 37°C and subsequently processed as previously described[7] to obtain free, membrane-bound and internalized fractions of the radioligand. The amount of radioactivity of each fraction was quantified in a γ-counter and calculated as percentage of the applied dose (n = 3 in triplicate).

Target-binding and internalization was also assessed for PC-3, LNCaP, and CHO-K1 after 2 h of incubation as a control. In analogy to the blocking experiments described above, potential mutual interferences were investigated by co-incubation of [^{111}In]In-RM2 with unlabeled PSMA-I&S (1000-fold) and [^{99m}Tc]Tc-PSMA-I&S with unlabeled RM2 (1000-fold). All cell fractions and 100 µL aliquots of radiolabeled peptides were measured in a γ-counter to calculate percentages of applied dose. The number of cells was determined using an automated cell counter in triplicates for each cell line. Data were normalized to 1x10^5 (LNCaP) or 1x10^6 cells (PC-3, CHO-K1, and transduced CHO-K1 cells), respectively.

All internalization experiments were carried out with radiolabeled peptides providing a radiochemical purity of ≥ 95% (radio-HPLC).

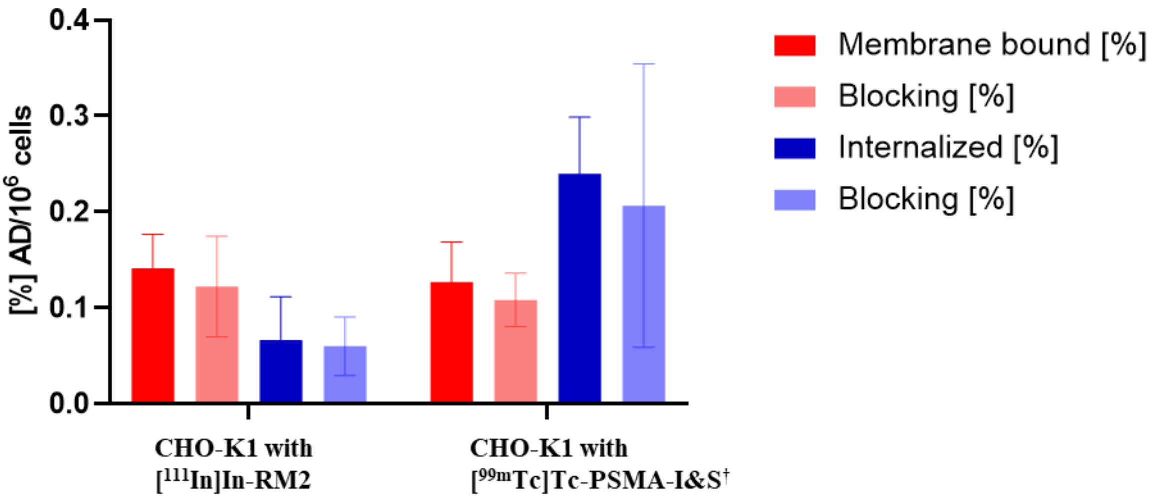

Figure S17. One time point (2 h) internalization assay with [^{111}In]In-RM2 and [^{99m}Tc]Tc-PSMA-I&S using CHO-K1 cells as a negative control. Non-specific binding (*Blocking*) was determined with 1000-fold excess of BBN(1–14) or 2-PMPA for [^{111}In]In-RM2 and [^{99m}Tc]Tc-PSMA-I&S, respectively. Data points show mean values ± SD (n = 3 and †n = 2 in triplicates). Data are normalized to 10^6 cells per well.

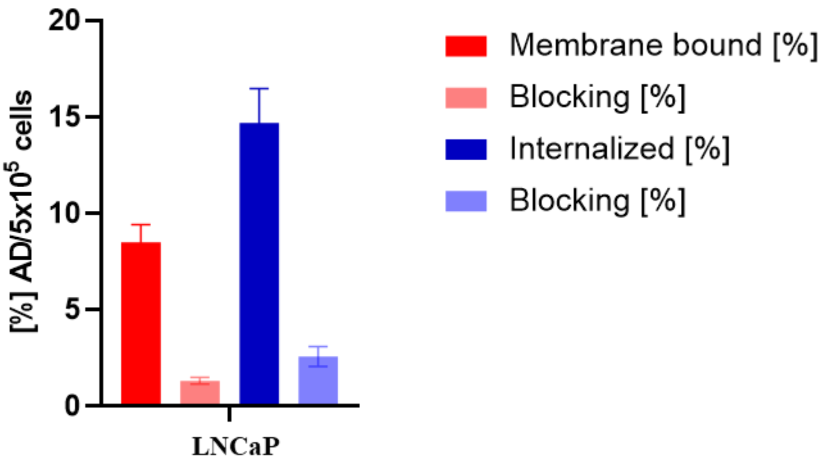

Figure S18. One time point (2 h) internalization assay with [^{99m}Tc]Tc-PSMA-I&S using LNCaP as a positive control. Non-specific binding (*Blocking*) was determined with 1000-fold excess of 2-PMPA. Data points show mean values ± SD (n = 3 in triplicate). Data are normalized to 5 x 10^5 cells per well.

3.5 γ-Counter set-up for dual isotope measurements

In order to estimate to what extent the later dual tracer biodistribution samples can be correctly quantified with regard to their ^{111}In and ^{99m}Tc content, samples with a predefined ^{99m}Tc and ^{111}In ratio were measured. The probes were measured for several times over a period of 2-3 weeks by an open window protocol (15 - 2000 keV, no decay correction, no background correction, 60 seconds per probe) and the data was processed as explained in section 3.6.1.1. Results are listed in *Table S1* and *Table S2*.

Table S1. Accuracy of ^{99m}Tc activity as determined by specific calculation methods based on continuous γ-counter measurements of ^{111}In/^{99m}Tc mixtures with predefined isotope ratios using an open window protocol. Values highlighted in red represent accuracies below 90%.

	^{99m}Tc activity content in isotope mixtures						
	100%	95%	75%	50%	25%	5%	0%
Activity amount	Accuracy [%] of calculated ^{99m}Tc-content in isotope mixtures with respect to the actual value						
50 kBq	98.9	97.4	97.7	97.9	98.6	81.3	-
5 kBq	97.4	97.4	98.6	>99	91.3	68.5	-

Table S2. Accuracy of ^{111}In activity as determined by specific calculation methods based on continuous γ-counter measurements of $^{111}In/^{99m}Tc$ mixtures with predefined isotope ratios using an open window protocol. Values highlighted in red represent accuracies below 90%.

	^{111}In activity content in isotope mixtures						
	100%	95%	75%	50%	25%	5%	0%
Activity amount	Accuracy [%] of calculated ^{111}In-content in isotope mixtures with respect to the actual value						
50 kBq	98.8	97.7	97.3	98.4	93.3	97.1	-
5 kBq	70.6	79.7	76.1	71.8	79.3	71.1	-

A drop of accuracy below 90% could be observed for a ^{99m}Tc activity content of 5% for both 5 kBq and 50 kBq as well as for the entire 5 kBq measurement series of ^{111}In. Nevertheless, we considered this measuring and evaluation protocol to be sufficient to analyze the biodistribution probes of this proof-of-concept study. In the case of more routine use, this γ-counter set-up requires certain optimizations for a more accurate calculation of the individual isotope contents, in particular for probes with low In-111 content.

3.6 *Ex vivo* experiments

3.6.1 Biodistribution studies

5.22 - 34.5 MBq (0.37 - 2.69 nmol) of [^{111}In]In-RM2 or 6.00 - 54.0 MBq (0.16 - 0.67 nmol) of [^{99m}Tc]Tc-PSMA-I&S or both (dual tracer cocktail) were injected into the tail vein of tumor xenograft-bearing female NOD SCID mice. They were sacrificed by cervical dislocation under isofluoran anesthesia at different time points *post injectionem* (p.i.) after the last imaging time point. Selected organs were removed, weighed and organ activities measured in a γ-counter. For animals in which a dual tracer cocktail was administered, the organs were measured for several times over a period of 2-3 weeks to use these data sets in specific calculation methods to obtain the correct activity for each individual radionuclide at the start of the measurement series. The energy resolution of the γ-counter (NaI-based detector) did not allow for nuclide-pure quantification. Hence, two consecutive measurements using narrow ^{99m}Tc (140.5 ± 10%) and ^{111}In (245 ± 10% keV; measurements in the ^{111}In 171 ± 10% keV energy window wouldn't make sense due to potential crosstalk from ^{99m}Tc) energy windows was considered as not sufficient, due to several reasons:

1) An initial measurement of the probe(s) in the ^{111}In 245 keV window will give correct results for the ^{111}In activity in the probe (CPM), <u>but</u>:
2) An initial measurement of the probe(s) in the ^{99m}Tc window will overestimate the ^{99m}Tc activity in the probe (due to crosstalk from ^{111}In 171 keV photopeak).

3) If we would measure the probe(s) by an open window (0 - 2000 keV) protocol and subtract the CPM values from ^{99m}Tc 140.5 keV window from the overall CPM, the ^{111}In activity would be partially overestimated, since we cannot correct for Compton scattering, Backscattering, and X-rays from lead shielding (up to 110 keV) originating from ^{99m}Tc photons. At the same time, the ^{111}In CPM would be partially underestimated due to subtraction of counts originating from ^{99m}Tc (crosstalk from ^{99m}Tc 140.5 keV photopeak). A calculation of the proportion of partial over- and underestimation is not possible, as the ^{99m}Tc ratio in the probe is not known.
4) If we would measure the probe(s) by an open window (0 - 2000 keV) protocol and subtract the CPM values from ^{111}In 245 keV and 171 keV window from the overall CPM, the ^{99m}Tc activity would be partially overestimated, since we cannot correct for Compton scattering, Backscattering, and X-rays from lead shielding (0 - ca. 150 keV) originating from ^{111}In photons. At the same time, the ^{99m}Tc CPM would be partially underestimated due to subtraction of counts originating from ^{111}In (crosstalk from ^{111}In 171 keV photopeak and ^{111}In-based scattering). A calculation of the proportion of partial over- and underestimation is not possible, as the ^{111}In ratio in the probe is not known.
5) Concerning delayed measurements, it's not possible to state for which probe the ^{99m}Tc activity already reached complete decay (= under detection limit of γ-counter) or in which probe ^{99m}Tc is still present, due to the unknown $^{99m}Tc/^{111}In$ ratio. In this scenario we also need to consider, that depending on the applied activity, 10 x $t_{1/2}(^{99m}Tc)$ is not sufficient for complete decay. If we would measure the probe(s) after 2.5 - 5 d, and assume complete decay of ^{99m}Tc ($t_{1/2}(^{99m}Tc)$ = 6.007 h[5] → 10 (20) x 6.007 h = 60 h (120 h) = 2.5 d (5 d)) and assume that only ^{111}In ($t_{1/2}(^{111}In)$ = 2.805 d[5]) is the remaining nuclide we would underestimate/loose the ^{111}In activity in probes, in which a very low amount of ^{111}In was present initially (directly after dissection).
6) Residual background activity (Bremsstrahlung) from ^{99g}Tc ($t_{1/2}$ = 2.1 x 10^5 y[5]; β^- decay to stable ^{99}Ru) can slightly adulterate the data measured at later time points. After ^{99m}Tc decay, the CPM values from ^{99g}Tc stay more or less the same, but since the initial ratio of $^{99m}Tc/^{111}In$ in the probe is not known, the amount of CPM originating from ^{99g}Tc (and summing up with CPMs from ^{111}In at later measuring time points) cannot be calculated and subtracted from ^{111}In-only values. Instead, an iterative calculation process, based on estimations about $^{99m}Tc/^{111}In$ ratios after a first data analysis could lead to an approximate CPM value originating from ^{99g}Tc, which could then be subtracted from ^{111}In CPMs. In this study we didn't include corrections for CPMs originating from ^{99g}Tc, since the measuring

period of 2 - 3 weeks occasionally didn't allow for a complete decay of ^{111}In (in case of high initial activities e.g. in the kidney). Besides, for the residual organs, the CPM values at very late time points were in the low two- to three-digit CPM range. However, residual ^{99g}Tc decay might need to be considered in studies with even lower activities applied.

7) Different detection efficiencies of the γ-counter for ^{111}In and ^{99m}Tc don't play a role in these measurements, since only CPM values are considered, no Bq values.

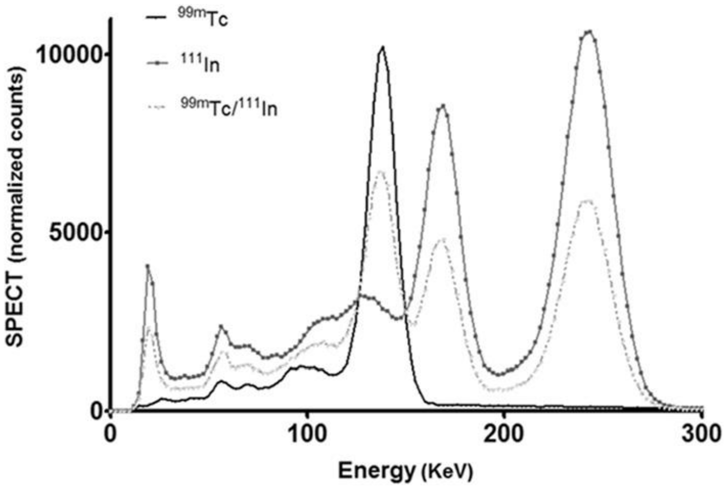

Figure S19. Energy spectra of ^{99m}Tc, ^{111}In and a mixture of both nuclides.[8] The image is depicted here to illustrate energy spectra as they appeared during γ-counter measurements of single and dual tracer probes. The image was taken from reference [8].

3.6.1.1 Calculation of A_0 for selected organs of dual tracer experiments

The following procedure is described exemplary for one organ (liver), but applies to all other dissected organs and body fluids (e.g. blood) as well.

In a first step the activities (CPM) of the multiple γ-counter measurements are listed (middle column, 'CPM - LIVER', *Table S3*) and a straight line is generated (*Figure S20*) based on *equation 5*, which can be obtained after applying the natural logarithm on the rearranged law of radioactive decay (*equation 2*). For ^{111}In, usually values after ^{99m}Tc decay (i.e. 10 x $t_{1/2}$(^{99m}Tc) = 60.07 h) are considered (≥ 5 data points, *Table S3*, highlighted in blue, ^{111}In decay dominant), with the time difference from the first measurement of this probe (Δt) plotted on the x-axis and the ln(A_t) value (≥ 5 data points, *Table S3*, highlighted in green) plotted on the y-axis.

$$A_t = A_0 \cdot e^{-\frac{\ln(2)}{t_{\frac{1}{2}}} \cdot t} \qquad (1)$$

$$\frac{A_t}{A_0} = e^{\frac{-\ln(2)}{t_{\frac{1}{2}}}\cdot t} \;\Big|\; ln \qquad (2)$$

$$\ln\left(\frac{A_t}{A_0}\right) = \frac{-\ln(2)}{t_{\frac{1}{2}}} \cdot t \qquad (3)$$

$$\ln(A_t) - \ln(A_0) = \frac{-\ln(2)}{t_{\frac{1}{2}}} \cdot t \qquad (4)$$

$$\ln(A_t) = \underbrace{-\frac{\ln(2)}{t_{\frac{1}{2}}}}_{\textbf{slope}} \cdot t + \underbrace{\ln(A_0)}_{\textbf{y-intercept}} \qquad (5)$$

Table S3: Exemplary table for the calculation of A_0(^{111}In) for any organ or body fluid in dual tracer experiments.

Δt	CPM - LIVER	ln(LIVER)	A_t In-111 (LIVER)	A_t Tc-99m (LIVER)	ln(A_t) Tc-99m (LIVER)
0,00	373640,14	12,83	**224134,142**	**149506,00**	11,91509179
1,12	353521,03	12,78	221567,8247	131953,2053	11,79020263
2,24	332998,19	12,72	219032,3641	113965,8259	11,64365391
3,35	316447,91	12,66	216525,6077	99922,30226	11,51214819
4,47	301718,21	12,62	214047,1424	87671,06765	11,38134722
5,59	289639,39	12,58	211596,9862	78042,40377	11,2650076
6,71	277239,83	12,53	209174,3679	68065,46213	11,1282252
7,82	266478,92	12,49	206779,2499	59699,67006	10,99708177
8,94	256801,84	12,46	204410,6212	52391,21883	10,86649428
10,06	248309,25	12,42	202068,9224	46240,32763	10,74160759
11,18	239337,34	12,39	199752,621	39584,71904	10,58619844
12,30	232896,22	12,36	197463,7751	35432,44492	10,4753832
13,42	225626,6	12,33	195201,3512	30425,24881	10,3230281
14,54	219244,35	12,30	192964,49	26279,86004	10,17655815
15,66	214000,27	12,27	190753,4252	23246,84478	10,05392469
16,78	209578,88	12,25	188567,1293	21011,75075	9,95283712
17,90	203560,73	12,22	186405,4112	17155,31875	9,750063536
19,01	200697,45	12,21	184268,6859	16428,76412	9,706788987
20,13	195778,75	12,18	182155,6716	13623,07841	9,519520575
21,25	192655,96	12,17	180066,707	12589,25302	9,440598794
22,37	187528,7	12,14	178000,7565	9527,943518	9,161984183
23,49	184248,37	12,12	175958,5092	8289,860847	9,022788462
24,62	181808,56	12,11	173939,1207	7869,43927	8,97074209
25,74	177390,79	12,09	171942,5143	5448,275733	8,603054458
50,13	133556,7	11,80	133747,8187		

69,27	108914,55	11,60	109806,4456
98,47	80714,49	11,30	81292,2
117,51	66472,63	11,10	66815,56539
146,05	49486,5	10,81	49796,153
168,06	39713,41	10,59	39696,13225
194,94	29891,6	10,31	30095,68754
220,78	22920,35	10,04	23061,55831
237,17	19454,2	9,88	19479,68763
266,87	14119	9,56	14346,35263
285,31	11873,58	9,38	11864,34691
313,68	8763,92	9,08	8858,4547
333,26	7109,28	8,87	7240,034345

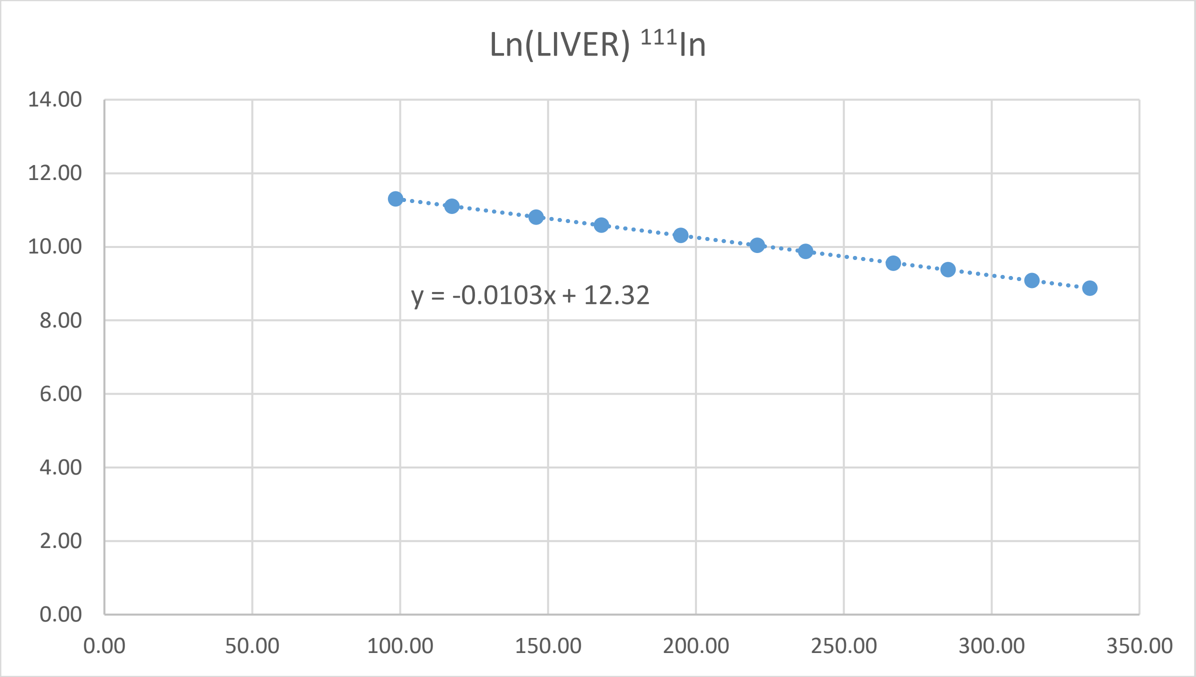

Figure S20: Straight line obtained by plotting the ln(organ, ^{111}In) values (y-axis) against Δt (x-axis; values after ^{99m}Tc decay, i.e. 10 x $t_{1/2}$(^{99m}Tc) = 60.07 h). The linear equation of this straight line can be used to calculate $A_{0,In\text{-}111}$ of the respective organ (*equations 7 and 8*).

The resulting linear equation provides a slope, which should be as close as possible to the theoretical slope, matching the half-life of ^{111}In (*equation 6*). The slope accuracy can be calculated by dividing the slope obtained by the linear equation by the theoretical slope and should be 100 ± 5%.

$$-\frac{\ln(2)}{t_{\frac{1}{2}}} = -\frac{\ln(2)}{2.805 \cdot 24\ \mathrm{h}} = -0.0103 \qquad (6)$$

Besides, the y-intercept will give the value of $\ln(A_0)$, since at t = 0, $\ln(A_t) = \ln(A_0)$ (*equation 5*). By applying the natural exponential function (inverse function of the natural logarithm) on the y-intercept, $A_{0,In\text{-}111}$ of the respective organ can be obtained (*equations 7 and 8*).

$$e^{\ln(A_0)} = A_0 \qquad (7)$$

$$A_{0,\mathrm{In-111}} = e^{12.32} = 224134.142 \qquad (8)$$

Subtraction of this value from the overall CPM value at $\Delta t = 0$ gives $A_{0,\ Tc\text{-}99m}$ (*equation 9*).

$$A_{0,Tc-99m} = A_{0,total} - A_{0,\mathrm{In-111}}$$

$$= 373640.14\ \mathrm{CPM} - 224134.14\ \mathrm{CPM} = 149506.00\ \mathrm{CPM} \qquad (9)$$

As a control, the $\ln(A_{t,Tc\text{-}99m})$ values can be calculated based on very early time points (^{99m}Tc decay dominant), e.g. from $\Delta t = 0$ - 10.06 h and plotted as before (*Figure S21*, respective values highlighted in gray and orange in *Table S3*). The resulting slope should give $-\ln(2)/t_{1/2}(^{99m}\mathrm{Tc})$ (= -0.1154) with an accuracy of 100 ± 5%.

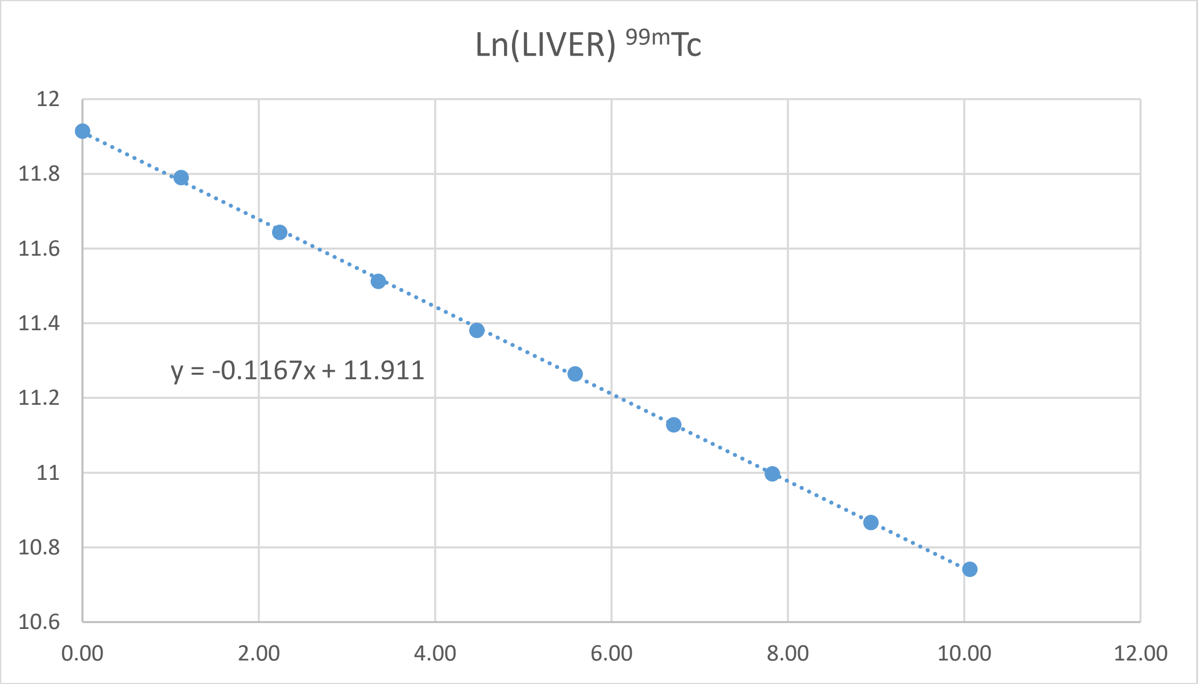

Figure S21: Straight line obtained by plotting the ln(organ, ^{99m}Tc) values (y-axis) against Δt (x-axis; values at very early measuring time points, e.g. from $\Delta t = 0$ - 10.06 h). The linear equation of this straight line can be used as a control to verify the $A_{0,Tc\text{-}99m}$ value obtained for the respective organ by *equation 9*.

The CPM values thus obtained for ^{111}In and ^{99m}Tc for each organ/body fluid were corrected to the time point at which the very first probe of the measurement series was measured. The CPM values were converted into % of injected dose (ID) based on the applied activity and normalized to the weight of the probe to obtain % ID/g, analogous to single tracer measurements.

The values used for generating *Figure 9* and *10* in the main manuscript are listed in Table *S4* and *S5*.

3.6.1.2 Biodistribution data of [^{111}In]In-RM2 and [^{99m}Tc]Tc-PSMA-I&S

Table S4: Biodistribution data of [^{111}In]In-RM2 at different time points, with different amounts of substance and different activities injected (5.22 – 34.5 MBq) in female tumor xenograft-bearing NOD SCID mice. Data are expressed as percentage of the injected dose per gram (% ID/g). Single Tracer: Only [^{111}In]In-RM2 was injected; Dual Tracer: [^{111}In]In-RM2 and [^{99m}Tc]Tc-PSMA-I&S were injected and values for [^{111}In]In-RM2 were determined as described in section 3.6.1.1. PC-3 xenograft: Mice bearing only PC-3 xenografts; Dual xenograft: Mice bearing both CHO-K1-GRPR and -PSMA xenografts. Biodistribution time points and amount of substances in brackets refer to [^{111}In]In-RM2.

Organ	**Mouse #1** Single tracer PC-3 xenograft (25.1 h p.i., 1.34 nmol)	**Mouse #2** Single tracer PC-3 xenograft (7.3 h p.i., 1.43 nmol)	**Mouse #3** Single tracer PC-3 xenograft (4 h p.i., 1.49 nmol)	**Mouse #4** Single tracer Dual xenograft (24.2 h p.i., 0.58 nmol)	**Mouse #5** Single tracer Dual xenograft (24.2 h p.i., 0.37 nmol)	**Mouse #6** Single tracer Dual xenograft (25.2 h p.i., 1.08 nmol)	**Mouse #7** Single tracer Dual xenograft (24.2 h p.i., 1.41 nmol)	**Mouse #8** Dual tracer Dual xenograft (25.8 h p.i., 1.00 nmol)	**Mouse #9** Dual tracer Dual xenograft (17.6 h p.i., 2.69 nmol)
**Blood**	n.d.	n.d.	0.03	n.d.	n.d.	0.01	0.01	0.02	0.02
**Heart**	0.03	0.02	0.02	0.02	0.03	0.03	0.02	0.00	0.03
**Lung**	0.05	0.03	0.05	0.03	0.03	0.05	0.04	0.04	0.03
**Liver**	0.17	0.12	0.22	0.10	0.13	0.20	0.17	0.28	0.13
**Spleen**	0.16	0.13	0.10	0.07	0.08	0.13	0.08	0.25	0.10
**Pancreas**	0.06	0.05	0.09	0.07	0.10	0.06	0.04	0.07	0.04
**Stomach (empty)**	0.05	0.02	0.09	0.03	0.04	0.04	0.03	0.04	0.03
**Intestine (empty)**	0.08	0.03	0.08	0.03	0.06	0.05	0.04	0.05	0.04
**Colon (empty)**	0.04	0.04	0.05	0.04	0.08	0.05	0.03	0.04	0.03
**Kidney**	2.17	1.29	2.20	1.14	1.42	1.69	1.34	1.07	1.09
**Adrenal**	0.18	0.49	0.13	0.12	0.33	0.16	0.08	0.03	0.12
**Muscle**	0.01	0.01	0.01	0.01	0.01	0.02	0.01	0.01	0.01
**Bone**	0.07	0.05	0.04	0.04	0.04	0.06	0.04	0.06	0.05
**CHO-K1-PSMA Tumor**	-	-	-	0.05	0.08	0.09	0.08	0.08	0.09
**CHO-K1-GRPR Tumor**	-	-	-	0.28	0.51	0.33	0.06	0.38	0.26
**PC-3 Tumor**	2.20	1.36	2.75	-	-	-	-	-	-

Table S5: Biodistribution data of [^{99m}Tc]Tc-PSMA-I&S at different time points, with different amounts of substance and different activities injected (6.00 – 54.0 MBq) in female tumor xenograft-bearing NOD SCID mice. Data are expressed as percentage of the injected dose per gram (% ID/g). Single Tracer: Only [^{99m}Tc]Tc-PSMA-I&S was injected; Dual Tracer: [^{99m}Tc]Tc-PSMA-I&S and [^{111}In]In-RM2 were injected and values for [^{99m}Tc]Tc-PSMA-I&S were determined as described in section 3.6.1.1. Dual xenograft: Mice bearing both CHO-K1-GRPR and -PSMA xenografts. Biodistribution time points and amount of substances in brackets refer to [^{99m}Tc]Tc-PSMA-I&S.

.

**Organ**	**Mouse #8** Dual tracer Dual xenograft (25.8 h p.i., 0.27 nmol)	**Mouse #9** Dual tracer Dual xenograft (17.6 h p.i., 0.16 nmol)	**Mouse #10** Single tracer Dual xenograft (25.1 h p.i., 0.28 nmol)	**Mouse #11** Single tracer Dual xenograft (25.6 h p.i., 0.36 nmol)	**Mouse #12** Single tracer Dual xenograft (5.2 h p.i., 0.45 nmol)	**Mouse #13** Single tracer Dual xenograft (6.6 h p.i., 0.67 nmol)
**Blood**	0.24	0.08	0.16	0.16	0.35	0.32
**Heart**	0.39	0.14	0.31	0.24	0.24	0.29
**Lung**	0.67	0.26	0.86	0.94	0.66	0.71
**Liver**	0.91	0.20	1.13	1.15	0.81	1.19
**Spleen**	2.37	0.36	2.53	2.95	5.72	4.94
**Pancreas**	0.34	0.14	0.29	0.45	0.32	0.37
**Stomach (empty)**	0.49	0.13	0.41	0.54	0.37	0.42
**Intestine (empty)**	0.46	0.11	0.26	0.24	0.40	0.35
**Colon (empty)**	0.31	0.13	0.48	0.58	0.54	0.44
**Kidney**	96.54	49.55	100.43	93.20	17.60	12.39
**Adrenal**	0.63	1.31	1.84	1.71	3.78	2.80
**Muscle**	0.24	0.03	0.17	0.08	0.09	0.10
**Bone**	2.74	0.68	2.34	3.13	2.30	1.77
**CHO-K1-PSMA Tumor**	2.45	0.44	2.08	1.37	3.51	2.02
**CHO-K1-GRPR Tumor**	0.60	0.12	0.61	0.47	0.53	0.46

3.6.2 Protein extraction and Western blot analysis of cell and tissue lysates

Cell pellets were dissolved in radioimmunoprecipitation assay (RIPA) buffer (#20-188, Merck) with the recommended amount of standard protease inhibitor cocktail (#P2714, Merck), frozen in liquid nitrogen, put at 37 °C to thaw, then frozen again and put on ice until completely thawed. The solution was centrifuged for 30 min at 4 °C and protein concentration of the supernatant was measured using a bicinchoninic acid (BCA) kit (#PR 23227, Thermo Scientific). Aliquots were stored at -80 °C in case of later use.

Tissue samples (CHO-K1-GRPR, CHO-K1-PSMA, LNCaP or PC-3 xenograft sections) were collected during dissection for biodistribution studies and stored at -80°C until no radioactivity could be detected anymore. Tissues were thawed on ice and extracted using an Ultra Turrax® tissue homogenizer (IKA, Germany) in the presence of RIPA buffer (#20-188, Merck) and the recommended amount of standard protease inhibitor cocktail (#P2714, Merck). The suspension was transferred in Eppendorf tubes and gently shaken at 4 °C for 30 min. After centrifugation (12 000 rpm, ~14,000 × g, 4 °C, 20 min), protein concentration in supernatants was determined with a BCA kit (#PR 23227, Thermo Scientific) and aliquots stored at -80 °C in case of later use.

For Western blotting, cell and tissue samples were diluted with 2× protein loading dye (100 mM Tris–HCl pH 6.8, 200 mM DTT, 4% SDS, 20% Glycerin, bromophenolblue) and RIPA Buffer with the recommended amount of standard protease inhibitor cocktail (#P2714, Merck) and heated for 5 min at 95 °C. 15 µg of protein were loaded per lane. Gel electrophoresis was performed on TGX™ precast gels (5-15% gradient, #456-1083, Bio-Rad), followed by semidry blotting on nitrocellulose membranes (#10600004, GE Healthcare Life Science) for 30 min using the TransBlot® Turbo™ Transfer System (Bio-Rad). After total protein staining (TPS) with Ponceau S (#P7170, Sigma-Aldrich) to ensure consistent protein loading, the membranes were blocked in 5% non-fat dry milk powder in 1X TBS-T (0.1% Tween 20) for 90 min at room temperature.

For detection of GRPR, a mouse anti-GRPR mAb (D-1, sc-398549 SCB, Santa Cruz Biotechnology, dilution 1:200 in 5% non-fat dry milk powder in 1X TBS-T (0.1% Tween 20)) was used as primary antibody and a rabbit anti-mouse pAb, HRP-conjugated (#315-035-008, Jackson ImmunoResearch, dilution 1:10 000 in 5% non-fat dry milk powder in 1X TBS-T (0.1% Tween 20)) was used as secondary antibody. Protein loading per pocket: 15 µg. (observed) Molecular weight of GRPR, detected by this primary mAb: ~ 90 kDa.

For detection of PSMA (*Figure 11,* main manuscript), a rabbit anti-PSMA mAb (D4S1F, CST, dilution 1:1000 in 5% non-fat dry milk powder in 1X TBS-T (0.1% Tween 20)) was used as

primary antibody and a goat anti-rabbit pAb, HRP-conjugated (A16104, Thermo Fisher Scientific, dilution 1:2500 in 5% non-fat dry milk powder in 1X TBS-T (0.1% Tween 20)) was used as secondary antibody. Protein loading per pocket: 15 µg. (observed) Molecular weight of PSMA, detected by this primary mAb: ~100 kDa.

The membranes were incubated with the respective primary antibody overnight at 4 °C. After washing thoroughly with TBS-T (3 x 7 min), incubation with the respective secondary antibody for 1 h at room temperature followed. After washing again with TBS-T, membranes were treated with the Clarity™ Western ECL substrate detection kit (#170-5060, Bio-Rad) and chemiluminescence signals were detected with the ChemiDoc™ Imaging System (Bio-Rad).

After detection of PSMA or GRPR signals, the membrane was incubated with Restore™ Western blot stripping buffer (Thermo Scientific #21059) for 30 min at room temperature and then prepared for incubation with a primary rabbit anti-β-actin pAb (ab8227, Abcam, dilution 1:2500 in 2% BSA in 1X TBS-T (0.1% Tween 20)) and a secondary goat anti-rabbit pAb, HRP-conjugated (A16104, Thermo Fisher Scientific, dilution 1:2500 in 2% BSA in 1X TBS-T (0.1% Tween 20)) and treated as described above. Protein loading per pocket: 15 µg. (observed) Molecular weight of β-actin, detected by this primary pAb: ~42 kDa. β-Actin Western blots were used as loading control.

4 ABBREVIATIONS

2-PMPA	2-Phosphonomethyl pentanedioic acid
ACN	Acetonitrile
BBN	Bombesin
BCA	Bicinchoninic acid
BSA	Bovine serum albumin
CHO	Chinese hamster ovary
CPM	Counts per minute
DIPEA	*N,N*-Diisopropylethylamine
DMEM	Dulbecco's Modified Eagle Medium
DMF	Dimethylformamide
DTT	Dithiothreitol
EDTA	Ethylenediaminetetraacetic acid
FBS	Fetal bovine serum
FOLH1	Folate hydrolase 1

GFP	Green fluorescent protein
GRPR	Gastrin-releasing peptide receptor
HATU	*O*-(7-azabenzotriazol-1-yl)-*N,N,N',N'*-tetramethyluronium hexafluorophosphate
HRP	Horseradish peroxidase
mAb	Monoclonal antibody
MBHA	4-Methylbenzhydrylamine
NMR	Nuclear magnetic resonance
NOD	Non-obese diabetic
pAb	Polyclonal antibody
PB	Phosphate buffer
p.i.	post injectionem
PSMA	Prostate-specific membrane antigen
RIPA	radioimmunoprecipitation assay
RNA	Ribonucleic acid
RP-HPLC	reversed-phase high performance liquid chromatography
RT-qPCR	Reverse transcription quantitative polymerase chain reaction
SCID	Severe combined immunodeficiency
SDS-PAGE	Sodium dodecyl sulfate polyacrylamide gel electrophoresis
SPPS	Solid-phase peptide synthesis
TBS-T	Tris-buffered saline with Tween 20
TCEP	Tris(2-carboxyethyl)phosphine
TFA	Trifluoroacetic acid
TIPS	Triisopropylsilane
TPS	Total protein staining
Tris	Tris(hydroxymethyl)aminomethane
UHR-TOF	Ultra-High-Resolution-Time-of-Flight

5 REFERENCES

1. Chan, W. and P. White, *Fmoc Solid Phase Peptide Synthesis: A Practical Approach*. 1999: Oxford University Press.
2. Mansi, R., et al., *Development of a potent DOTA-conjugated bombesin antagonist for targeting GRPr-positive tumours.* Eur J Nucl Med Mol Imaging, 2011. **38**(1): p. 97-107.
3. Robu, S., et al., *Preclinical Evaluation and First Patient Application of 99mTc-PSMA-I&S for SPECT Imaging and Radioguided Surgery in Prostate Cancer.* Journal of Nuclear Medicine, 2017. **58**(2): p. 235-242.
4. Konrad, M., et al., *[99mTc]Tc-PentixaTec: development, extensive pre-clinical evaluation, and first human experience.* European Journal of Nuclear Medicine and Molecular Imaging, 2023. **50**(13): p. 3937-3948.
5. *https://www-nds.iaea.org/relnsd/vcharthtml/VChartHTML.html*. accessed on January 04, 2024 .
6. Hassler, M.R., et al., *Antineoplastic activity of the DNA methyltransferase inhibitor 5-aza-2'-deoxycytidine in anaplastic large cell lymphoma.* Biochimie, 2012. **94**(11): p. 2297-2307.
7. Giammei, C., et al., *Sorbitol as a Polar Pharmacological Modifier to Enhance the Hydrophilicity of 99mTc-Tricarbonyl-Based Radiopharmaceuticals.* Molecules, 2020. **25**(11): p. 2680.
8. Hendrikx, G., et al., *Molecular imaging of angiogenesis after myocardial infarction by (111)In-DTPA-cNGR and (99m)Tc-sestamibi dual-isotope myocardial SPECT.* EJNMMI Res, 2015. **5**: p. 2.
